# A novel deep learning approach for typhoon-induced storm surge modeling through efficient emulation of wind and pressure fields

**DOI:** 10.1038/s41598-023-35093-9

**Published:** 2023-05-16

**Authors:** Iyan E. Mulia, Naonori Ueda, Takemasa Miyoshi, Takumu Iwamoto, Mohammad Heidarzadeh

**Affiliations:** 1 Prediction Science Laboratory, RIKEN Cluster for Pioneering Research, Kobe, Japan; 2 Disaster Resilience Science Team, RIKEN Center for Advanced Intelligence Project, Tokyo, Japan; 3 Data Assimilation Research Team, RIKEN Center for Computational Science, Kobe, Japan; 4 Tsunami and Storm Surge Research Group, Port and Airport Research Institute, Yokosuka, Japan; 5 Department of Architecture and Civil Engineering, University of Bath, Bath, BA2 7AY UK

**Keywords:** Natural hazards, Ocean sciences

## Abstract

Modeling typhoon-induced storm surges requires 10-m wind and sea level pressure fields as forcings, commonly obtained using parametric models or a fully dynamical simulation by numerical weather prediction (NWP) models. The parametric models are generally less accurate than the full-physics models of the NWP, but they are often preferred owing to their computational efficiency facilitating rapid uncertainty quantification. Here, we propose using a deep learning method based on generative adversarial networks (GAN) to translate the parametric model outputs into a more realistic atmospheric forcings structure resembling the NWP model results. Additionally, we introduce lead-lag parameters to incorporate a forecasting feature in our model. Thirty-four historical typhoon events from 1981 to 2012 are selected to train the GAN, followed by storm surge simulations for the four most recent events. The proposed method efficiently transforms the parametric model into realistic forcing fields by a standard desktop computer within a few seconds. The results show that the storm surge model accuracy with forcings generated by GAN is comparable to that of the NWP model and outperforms the parametric model. Our novel GAN model offers an alternative for rapid storm forecasting and can potentially combine varied data, such as those from satellite images, to improve the forecasts further.

## Introduction

The destructive force of typhoons impacting coastal areas is mainly attributed to the accompanying impacts from waves and storm surges^1^, possibly increasing future severity due to coastal population growth and climate change effects on the ocean and atmosphere^2–4^. Efforts have been made globally to mitigate the disaster, one of which is reflected in advancements in storm surge numerical models^5^. There are many successful applications of storm surge modeling to hindcast notable historical events^6–9^ revealing the individual catastrophe characteristics. Storm surge models can also be implemented as an integral part of operational forecasting systems^10,11^. Further enhancements of the present state-of-the-art models for storm surge simulations are expected to lean towards computational frameworks as the physical understandings of such a natural phenomenon have relatively matured^5^.

Storm surge models rely on wind and pressure fields acting as forcings for the hydrodynamic modeling of surge propagation and runup in coastal areas. A straightforward way to obtain these forcings is to use parametric models of typhoons, namely parameterized statistical formulas derived from past observations^12–14^. Parametric models have been reported to work well for storm surge simulations^15,16^. Nevertheless, poor predictive skills are typically exhibited in areas far from the typhoon center^8,17^ or during the typhoon transitioning stage into an extratropical cyclone and landfall due to topographic effects^18^. Similarly, standard mesoscale NWP models also exhibit some drawbacks partly caused by the insufficient grid resolutions for resolving a typhoon’s intensity^19^. Recent atmospheric and computer science developments have led to substantial improvements in the NWP models using a finer NWP model grid resolution^20^ or a data assimilation scheme^21^ to improve the typhoon structure resolvability. Therefore, many recent studies advocated using NWP models for storm surge simulations^8,9,17,22^.

The remaining issue of the NWP model implementation in storm surge modeling is related to the computational cost, which is significantly higher than that required for the parametric models. Furthermore, an ensemble storm surge prediction from multiple simulations is often preferred to provide a range of possible solutions rather than a single predicted value, thus facilitating uncertainty quantification^9,17^. The ensemble modeling approach will inevitably incur more computational efforts. Such obstacles restrict the realization of NWP models in regions with limited computational resources, particularly when the storm surge model is needed for an operational forecasting system. As an alternate solution, here we propose a method based on deep learning known for its computational efficiency compared to physics-based models. It has recently gained more attention and has been implemented in numerous geophysical applications^23–25^.

Deep learning or machine learning with various architectural building blocks has also been specifically adopted in storm surge-related studies^26–30^. Generally, these studies consider atmospheric and oceanic variables as predictors for the peak or time series of surges at specified points of interest on regional and global scales. Such a model configuration would benefit operational forecasting systems as it can provide accurate and rapid storm surge estimates, albeit it does not simulate a spatiotemporal evolution of storm surges. Thus, we explore a different approach for simulating storm surges focusing on the efficient emulation of forcing fields. This is because the dynamical atmospheric model is, to a large extent, the computationally demanding portion in the storm surge modeling compared to its hydrodynamics counterpart. The rationale of our approach is that the method can be used to study the physics of hydrodynamic responses to typhoons through a standard numerical simulation as well as to operate effectively in a forecasting mode. Our approach can also be useful for long-term risk assessment, typically estimated using statistical typhoon models^31^.

## Materials and methods

### Data acquisition

**Figure 1:**
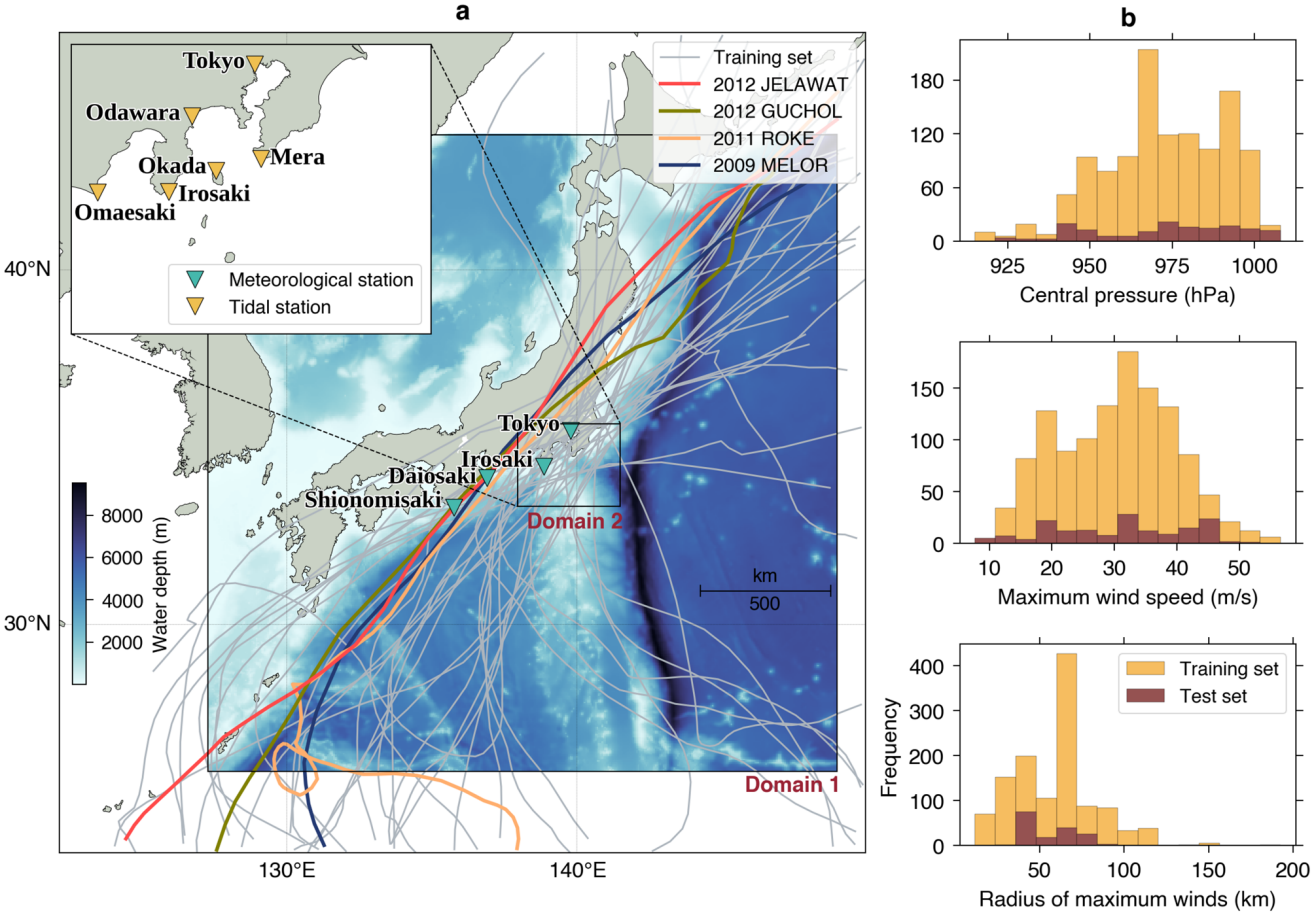
(**a**) NWP and parametric models domain and typhoon tracks in the training and test sets. Nested layers of the storm surge model are annotated by Domain 1 and 2, overlaid with water depth and observation stations. (**b**) Distributions of central pressure (top), maximum wind speed (mid), and radius of maximum winds (bottom) in the training and test sets. The map was created using the Matplotlib Basemap Toolkit (https://matplotlib.org/basemap/) in Python.

We obtain typhoon best track data required as inputs for the parametric model from the International Best Track Archive for Climate Stewardship (IBTrACS)^32,33^ and resample the data at one-hour intervals to pair with the NWP model outputs. Here, we refer to the NWP model to the Japanese 55-year Reanalysis (JRA-55)^34^ downscaled to a 5-km horizontal resolution named the Dynamical Regional Downscaling Using the JRA-55 Reanalysis (DSJRA-55)^35^ provided by the Japan Meteorological Agency (JMA). The reanalysis was conducted using a state-of-the-art data assimilation method incorporating various observational datasets overlooked in the operational system. Moreover, typhoon bogus fields from best track data were assimilated in the JRA-55^34^. Therefore, it yields the NWP model datasets that better resolve the typhoon’s intensity and track. We limit the area of the NWP model to fit our preferred domain depicted in Fig. 1a and select a period at which the IBTrACS and DSJRA-55 datasets overlap, that is, between 1981 and 2012. To validate the atmospheric model results, we use observed wind speed and sea level pressure at four meteorological stations administered by the Japan Oceanographic Data Center (JODC) with station locations marked in Fig. 1a.

The bathymetry for our storm surge simulations is based on two datasets: the General Bathymetric Chart of the Oceans (GEBCO_2020 Grid) with the original 15 arc-sec grid resolution for the open ocean (Domain 1 in Fig. 1a) and the Japan Hydrographic Association’s M7001 bathymetric contours for the nearshore region (Domain 2 in Fig. 1a). The overview of water depth profile from the above bathymetry data in our storm surge modeling domain is shown in Fig. 1a. The storm surge model is concentrated around Tokyo Bay, a crucial water body near the capital. Therefore, we compare our simulations with observed storm surges at six tide gauges in the vicinity of Tokyo Bay (see inset in Fig. 1a for locations) managed by the JMA. The surge is extracted from the observed water level by subtracting the predicted astronomical tide that the JMA also provides.

### Parametric model

While the NWP model is based on the precomputed reanalysis, we run the parametric model for the considered typhoon events using the Holland 1980 formula^12^, which has been widely adopted in storm surge modeling^15,16,36^. The inputs for this model along the typhoon tracks and durations are the central pressure, the maximum wind speed, and the radius of maximum winds (*R*_max_). Most input information is generally available in the IBTrACS dataset except for the *R*_max_. We impute the missing values of *R*_max_ in certain typhoon events using a technique proposed by Takagi and Wu^37^ derived from the radius of the 50 kt wind (*R*_50_). A surface wind reduction factor of 0.9 is applied to adjust the wind fields to a 10-m height wind^31^. The domain boundary (Fig. 1a) and grid size (5 km) for the parametric model are the same as the predetermined NWP model resulting in a square domain consisting of 512 × 512 grid points. The exact dimension for the NWP and the parametric models is intended for ease of transformation by deep learning.

### Deep learning

We split the simulated hourly data from pairs of the parametric and NWP models into a training set consisting of typhoon events during the period 1981–2009 (34 events) and a test set comprising the four latest events in the dataset that are: the 2009 Typhoon Melor, the 2011 Typhoon Roke, the 2012 Typhoon Guchol, and the 2012 Typhoon Jelawat. The complete list of typhoon events with their respective simulation times considered in this study is tabulated in Supplementary Table 1. The total number of data in the training set is 3478, and it is 457 in the test set. The training and test sets distributions are shown in Fig. 1a for the typhoon tracks and in Fig. 1b for central pressure, maximum wind speed, and radius of maximum winds. These distributions suggest that the test set is within the scope of the training set, which is necessary for deep learning or machine learning methods that commonly do not possess an extrapolation property.

**Figure 2:**
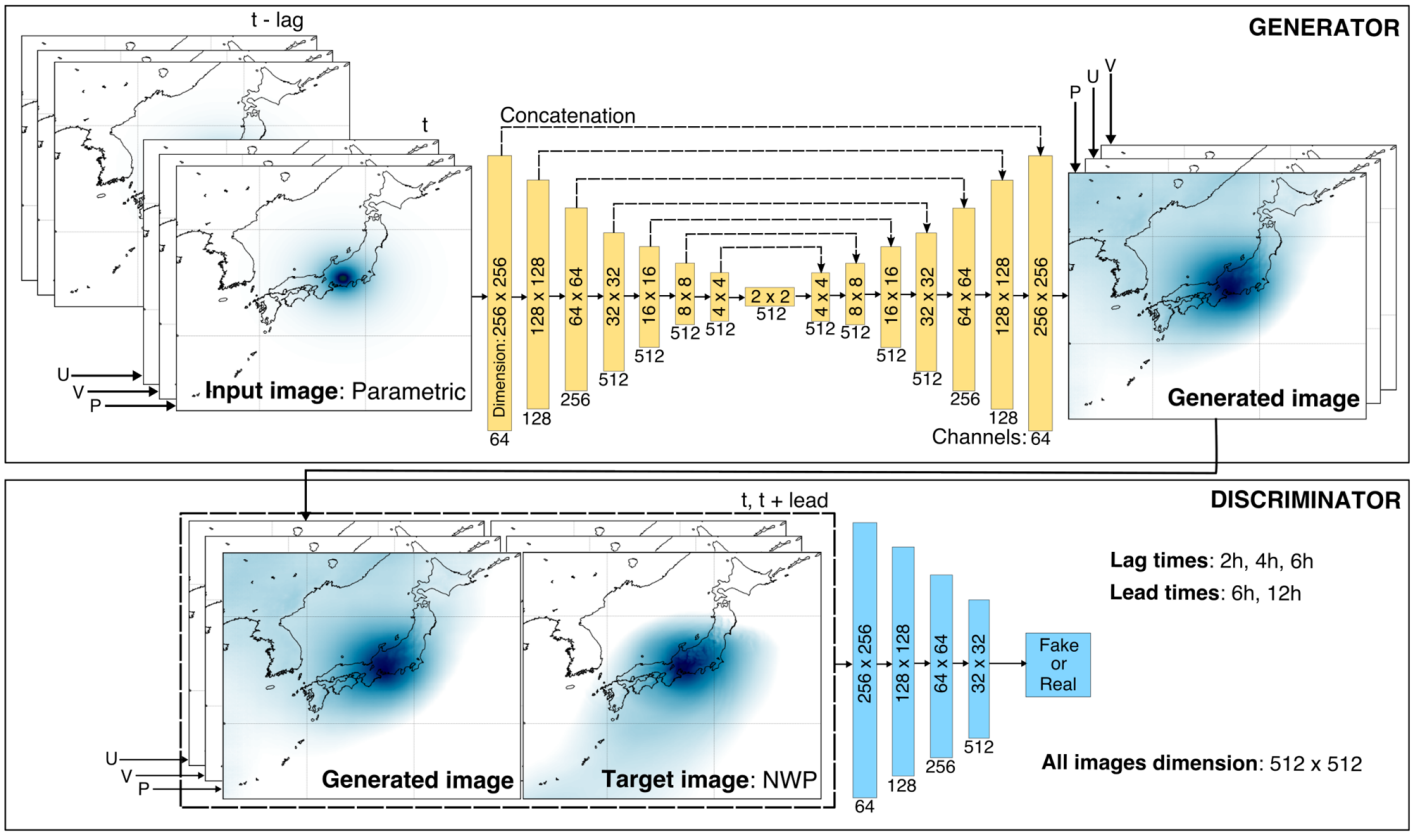
The schematic and architecture of the proposed GAN model. The input consists of atmospheric forcings from the parametric model with the specified lag times. The output is atmospheric forcings from the NWP model with a single time instance at *t*, *t* + 6 h, and *t* + 12 h. The figure was created using the Matplotlib Basemap Toolkit (https://matplotlib.org/basemap/) in Python and Inkscape (https://inkscape.org/).

We implement a deep learning method based on generative adversarial networks (GAN)^38^, constituting a generator to produce synthetic images and a discriminator to distinguish between real or target and the generated images. More specifically, we utilize a variant of GAN known as pix2pix developed for image-to-image translation problems^39^. The pix2pix algorithm is essentially a conditional GAN, where the output is conditional on the given input values instead of random latent space as in the initially-proposed GAN^38^. The generator in the pix2pix is taken from the U-Net, a U-shaped encoder-decoder network architecture^40^, while the discriminator is based on the PatchGAN classifier penalizing structure at the scale of local image patches^41^. This study uses the same architecture and hyperparameters as the original pix2pix algorithm^39^. However, we introduce a specific input–output configuration by incorporating lead-lag parameters that suit our application. For conciseness, hereafter, we refer to our deep learning method as GAN. The schematic of our GAN model is depicted in Fig. 2.

Our GAN input channels are two-dimensional fields of pressure (P) and wind components in eastward (U) and northward (V) directions from the parametric model with a dimension of 512 × 512 and a grid size of 5 km. Moreover, the input channels are extended to account for the lag times specified at two hours, four hours, and six hours. For instance, inputs for the 4-h lag time contain forcing fields (P, U, and V) from *t* to *t* − 4 h resulting in 15 channels. The optimal lag time is determined before applying the proposed GAN model to the storm surge simulations, discussed in detail in the subsequent section. Correspondingly, the output or target consists of forcing fields (P, U, and V) from the NWP model, with the same dimension and grid size as the parametric model. However, unlike the input, the output has three channels fixed to represent a single instance of time. Therefore, multiple models are built for different lead times. We design our model to perform nowcasting at time *t* and forecasting with two lead times at *t* + *6* h and *t* + 12 h*.* After normalizing the data to (− 1; 1), each model is trained for 200 epochs, and as a typical GAN model, only the generator is needed to make predictions once the training is complete.

### Determination of lead and lag times

**Figure 3:**
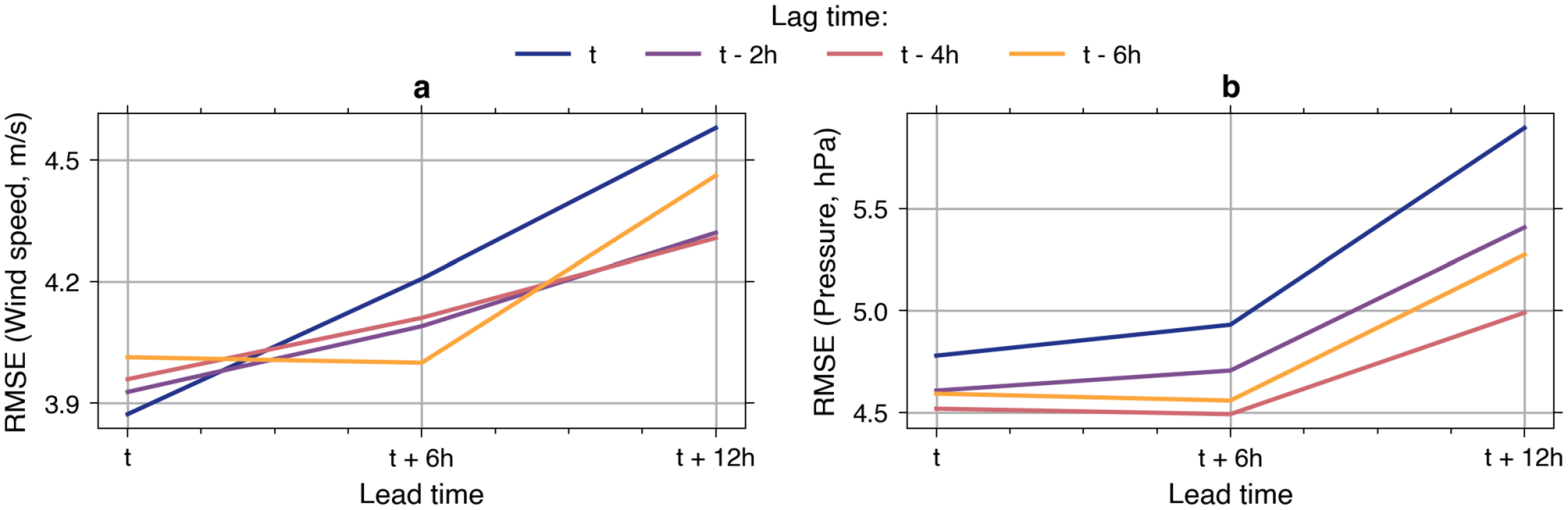
Model errors relative to lead and lag times. The RMSE is calculated from simulated versus observed time series of forcings at all meteorological stations. (**a**) Wind speed. (**b**) Sea level pressure.

We create a suite of GAN models with different lead and lag times combinations. The experiment aims at finding the optimal lag time, an important hyperparameter in forecasting models^42^. Using the test set, we calculate a root mean square error (RMSE) of simulated against observed time series of wind and sea level pressure at all considered meteorological stations. Figure 3 shows the RMSE comparison between the combinatorial models. It is difficult to determine a single lag time with consistent predictive skills from the wind errors (Fig. 3a). For example, a 4-h lag time exhibits the smallest RMSE only for the 12-h lead time. However, the error variations are arguably negligible for the overall lag times relative to the maximum winds in the test set (see the mid panel of Fig. 1b). Contrarily, from the sea level pressure errors (Fig. 3b), it is evident that the lag time of four hours results in the smallest RMSE for all lead times. Therefore, we opt for the lag time of four hours as the optimal value for our GAN model.

### Storm surge modeling

To simulate the storm surges, we employ a free-surface and terrain-following coordinate model known as the Regional Ocean Modeling System (ROMS)^43^. Previous studies have demonstrated the efficacy of ROMS in storm surge modeling^7,44,45^. Here, the model is configured to only account for the surge dynamics during typhoon occurrences without considering the interaction with tides and wind waves. We use a two-layer nested grid system as indicated in Fig. 1a with grid sizes of 5 km and 1 km for the parent (Domain 1 in Fig. 1a) and child (Domain 2 in Fig. 1a) domains, respectively. Similar to a study by Heidarzadeh et al.^7^, three vertical layers are used in our storm surge simulations. We set a minimum depth of 1 m because our model does not account for the inundation and the horizontal viscosity at 1000 m^2^/s. To convert the wind speed to the wind stress, we use the bulk flux module implemented in ROMS based on the Coupled Ocean–Atmosphere Response Experiment (COARE 3.0) algorithm^46^. The time integration of ROMS requires a decomposition of 3D fields into baroclinic and barotropic parts to facilitate the calculation of the pressure-gradient force. Thus, we set the simulation time steps for the parent and child grids at 45 s and 15 s in the baroclinic mode and 4.5 s and 1.5 s in the barotropic mode. For comparisons, we run the storm surge simulations for the four selected typhoon events in the test set using atmospheric forcings from the parametric, NWP, and GAN models.

## Results and discussion

### Simulated wind and pressure fields

**Figure 4:**
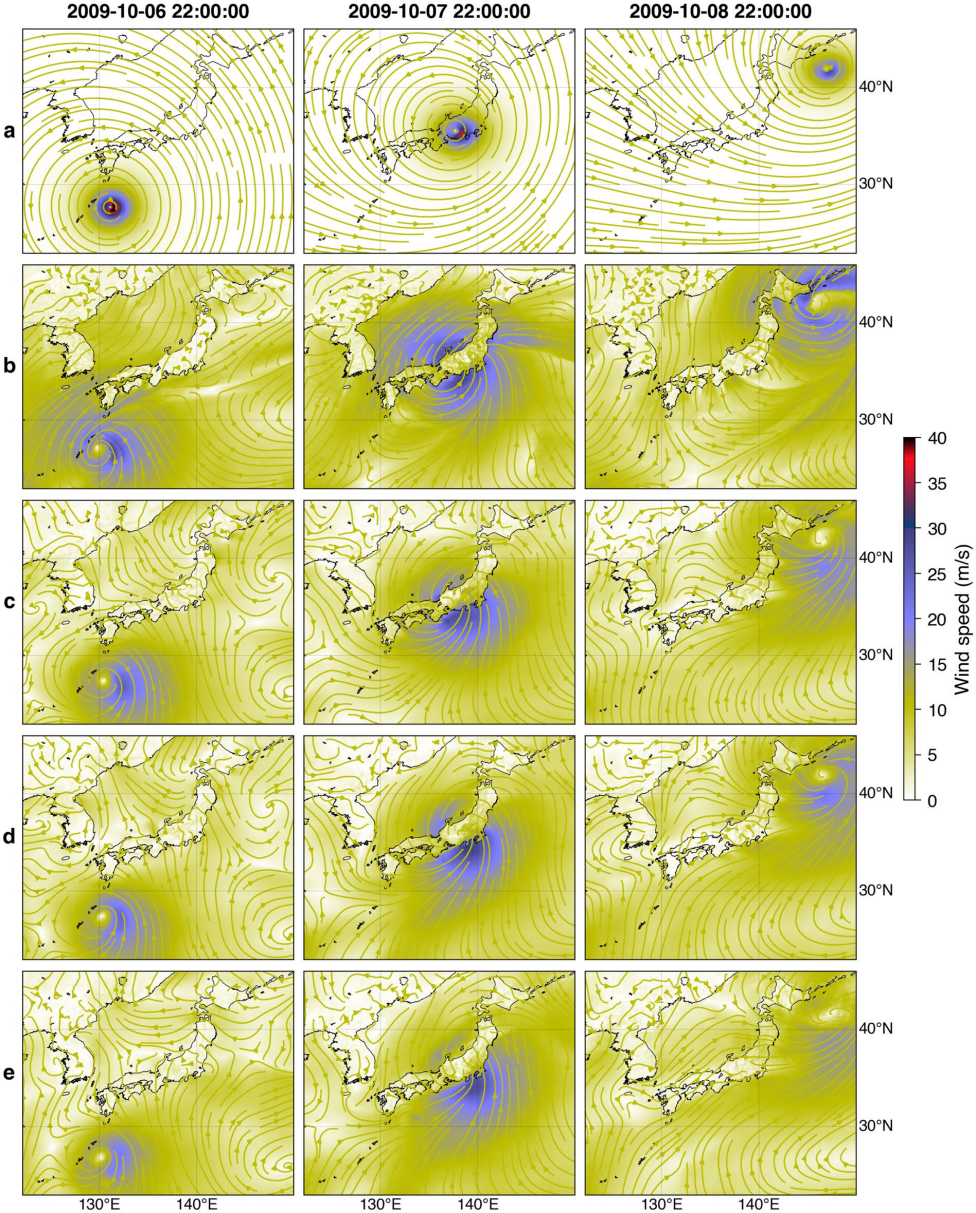
Snapshots of simulated wind fields of the 2009 Typhoon Melor using the parametric model (**a**), the NWP model (**b**), and GAN models at *t* (**c**), *t* + 6 h (**d**), and *t* + 12 h (**e**). The figure was created using the Matplotlib Basemap Toolkit (https://matplotlib.org/basemap/) in Python.

Figure 4 shows snapshots of simulated wind fields for the 2009 Typhoon Melor by the parametric model (Fig. 4a), the NWP model (Fig. 4b), and GAN models (Fig. 4c–e) at different lead times. Here, the parametric and the NWP models are not meant for forecasting, comparable to the GAN model at *t* in Fig. 4c. GAN models reasonably approximate the wind fields of the NWP model and maintain consistent structural patterns over different lead times. The topographic effects on the wind are well-captured by the GAN models characterized by a decrease in wind speed over land. Furthermore, the proposed GAN models can also somewhat simulate the typhoon transitioning stage into an extratropical cyclone, which the conventional parametric model hardly achieves. Nevertheless, the GAN models do not adequately simulate the north-easterly wind, an essential factor for simulating storm surges in the northern part of Japan. Also, the decreasing accuracy is expected at longer lead times, as exemplified in Fig. 3. Similar results are manifested in other typhoon events on the test set (Supplementary Figs. 1–3).

**Figure 5:**
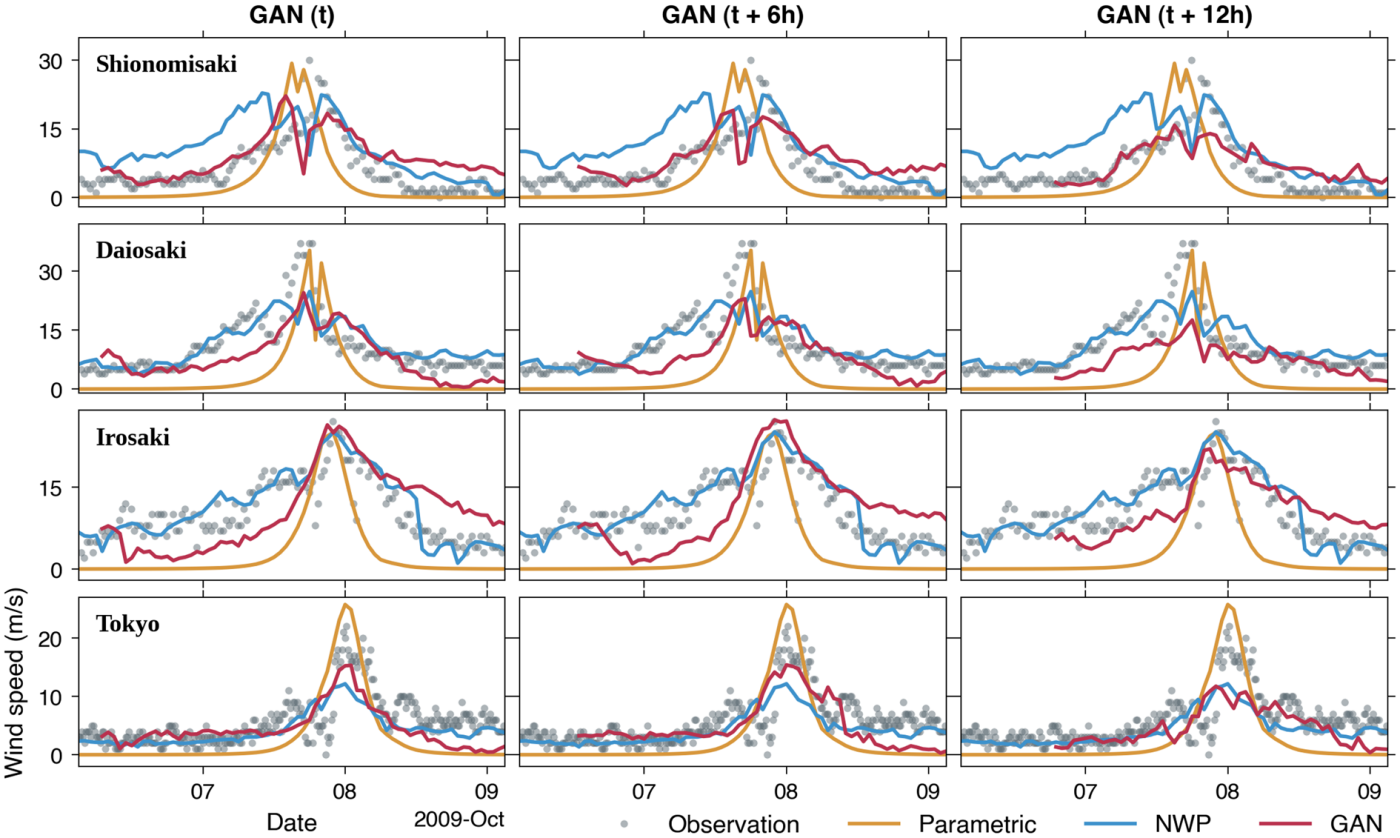
Comparisons between observed and simulated wind speed of the 2009 Typhoon Melor by the parametric model, the NWP model, and GAN models at *t*, *t* + 6 h, and *t* + 12 h.

Comparisons between observed and simulated wind speed at the considered meteorological stations are shown in Fig. 5. The parametric model accurately predicts the peaks of wind speed at most stations ranging from 22 to 37 m/s with a modest overestimation exhibited at Tokyo station. This overestimation tendency is also visible at other events and stations (Supplementary Figs. 4–6), which is in line with previous studies^17,47^. For the 2011 Typhoon Roke (Supplementary Fig. 4) at Tokyo station, the parametric model even results in a peak of wind speed of ~ 34 m/s, more than 1.5 times higher than the observation of 21 m/s. On top of that, the nature of the parametric model hinders a detailed approximation of small-scale variations in the observed wind time series. Accordingly, the simulated winds by the parametric model have smoother profiles with rather short periods compared to observations. The NWP model, on the other hand, shows better details and a wider spectrum of wave periods but tends to underestimate the peaks slightly. The GAN models would naturally inherit the characteristic of the NWP model as per the intended design, albeit the level of agreement varies for different stations and events (Supplementary Figs. 4–6).

**Figure 6:**
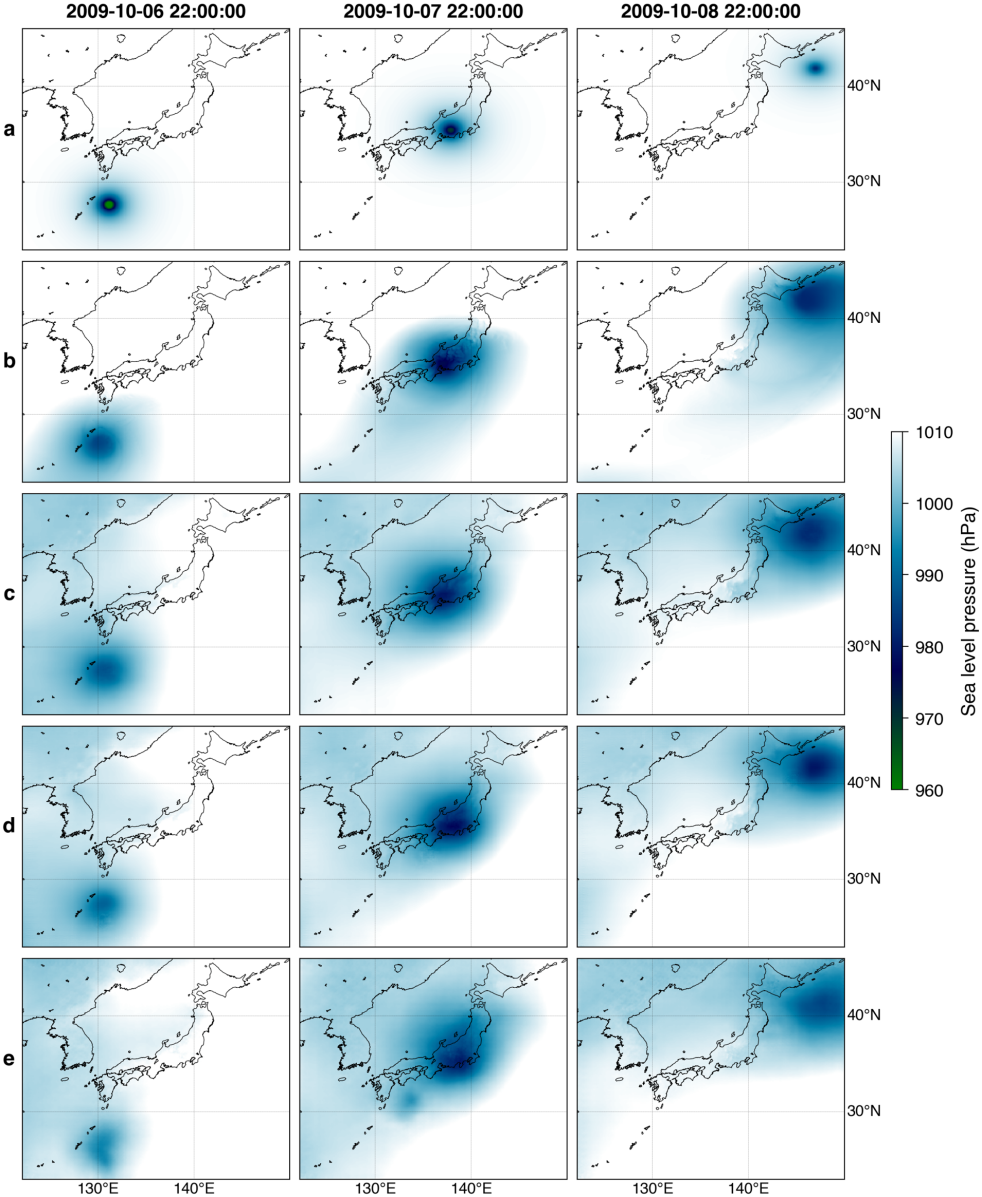
Snapshots of simulated sea level pressure fields of the 2009 Typhoon Melor using the parametric model (**a**), the NWP model (**b**), and GAN models at *t* (**c**), *t* + 6 h (**d**), and *t* + 12 h (**e**). The figure was created using the Matplotlib Basemap Toolkit (https://matplotlib.org/basemap/) in Python.

The snapshots of simulated sea level pressure fields for the 2009 Typhoon Melor are shown in Fig. 6. Additionally, an animation of simulated sea level pressure and wind fields for this event from all the models is provided in Supplementary Video 1. The spatial distribution of sea level pressure by the parametric model (Fig. 6a) shows simpler shapes than the other models with significant low-pressure values confined near the typhoon center. In contrast, the NWP model results in a relatively dispersed pressure distribution (Fig. 6b), which is physically more plausible. To some degree, GAN models for different lead times (Fig. 6c–e) replicate the NWP pressure fields, particularly around the typhoon’s inner core structure. There are somewhat erroneous low-pressure values northwest of the typhoon center, also found in other events (Supplementary Figs. 7–9). This is likely caused by the generalization of patterns captured in the training set. However, such a spurious occurrence is insignificant and has minimal effects on the corresponding storm surge.

**Figure 7:**
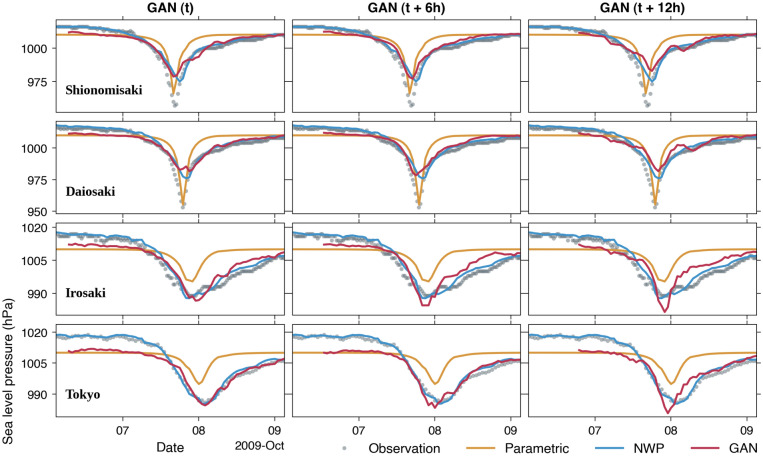
Comparisons between observed and simulated sea level pressure of the 2009 Typhoon Melor by the parametric model, the NWP model, and GAN models at *t*, *t* + 6 h, and *t* + 12 h.

We plot the time series comparisons between observed and simulated sea level pressure in Fig. 7. The simulated sea level pressure by the parametric model at Shionomisaki and Daiosaki is quite accurate. However, the model fails to predict the lowest pressure values of 989 hPa and 986 hPa at Irosaki and Tokyo, respectively. The poor parametric model’s performance is possibly related to the farther distances of the latter two stations to the typhoon center than the former stations. As can be seen from the typhoon tracks (Fig. 1), the relative typhoon track-to-stations distance for the remaining events in the test set is almost similar to the 2009 Typhoon Melor event. Therefore, the resemblance of predictive skills for other events is anticipated (Supplementary Figs. 10–12). The overall GAN models for various lead times mimic the NWP model results, especially near the primary curve of lowest pressure values at all stations, which play a pivotal role in the storm surge model accuracy.

### Simulated storm surges

**Figure 8:**
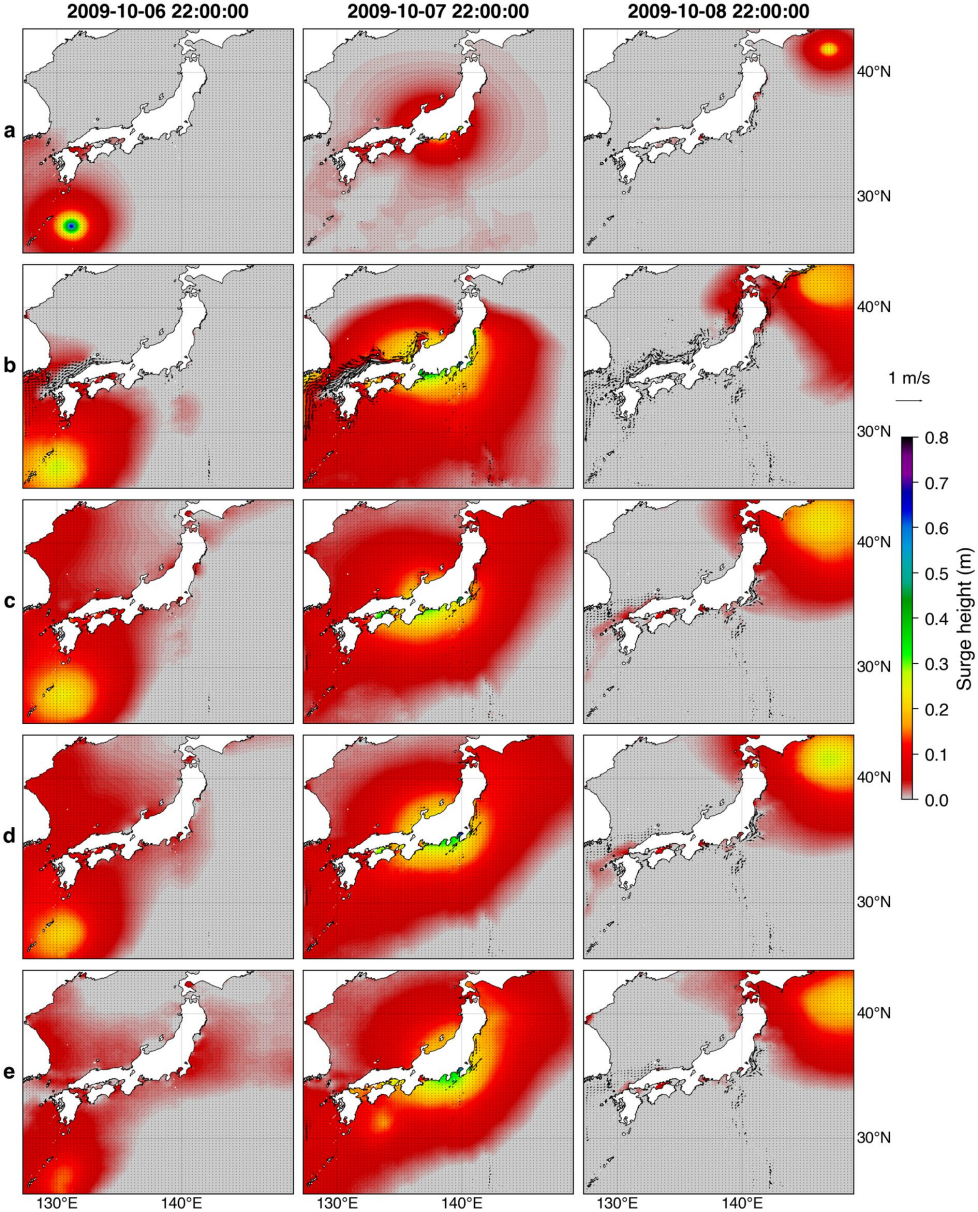
Snapshots of simulated storm surge and depth-averaged ocean currents during the 2009 Typhoon Melor using forcings from the parametric model (**a**), the NWP model (**b**), and GAN models at *t* (**c**), *t* + 6 h (**d**), and *t* + 12 h (**e**). The figure was created using the Matplotlib Basemap Toolkit (https://matplotlib.org/basemap/) in Python.

Figure 8 shows snapshots of the simulated storm surge during the 2009 Typhoon Melor. The entire storm surge simulations for this event using forcings from parametric, NWP, and GAN models with a one-hour interval are presented in a video file (Supplementary Video 2). From the atmospheric modeling results, it is foreseen that the corresponding storm surges with forcings from the NWP (Fig. 8b) and GAN models (Fig. 8c–e) would produce more realistic hydrodynamics responses than that of the parametric model (Fig. 8a). As an illustration, a bulge of sea surface caused by the tangential wind stress and inverse barometric effects covering appreciable surge heights of more than ~ 0.2 m using the NWP model generally matches the GAN model results at *t* (Fig. 8c) and *t* + 6 h (Fig. 8d). The extent of this high water surface elevation level propagating along the typhoon track correlated with the scale of the weather system by the parametric model is much smaller than both the NWP and GAN models. This comparison result, in conjunction with the inclusion of the forecasting feature, may highlight the advantage of GAN models in emulating the forcing fields.

However, despite the satisfactory performance in general, the method is still subjected to several limitations. A notable discrepancy is apparent at the early stage of simulation with forcings from GAN results with the 12-h lead time (Fig. 8e). Nonetheless, it has a minor influence on the surge prediction in the focused area near Tokyo Bay. Another limitation attributed to forcings from the GAN model for the 2009 Typhoon Melor is the lack of wind stress around the west coast of Japan correlated with the wind speed at the northwest sector of the typhoon’s inner core (see Fig. 4). Consequently, the storm surge model is unable to reproduce the strong ocean currents in the range ~ 0.5–0.9 m/s simulated using forcings from the NWP model. The same result is seen for the 2011 Typhoon Roke (Supplementary Fig. 13), but not for the 2012 Typhoons Guchol (Supplementary Fig. 14) and Jelawat (Supplementary Fig. 15), suggesting that the error is not systematic. It implies that many typhoon events in the training set have asymmetric wind profiles dominated by strong winds in the southeast vortex analogous to the 2012 Typhoons Guchol and Jelawat.

**Figure 9:**
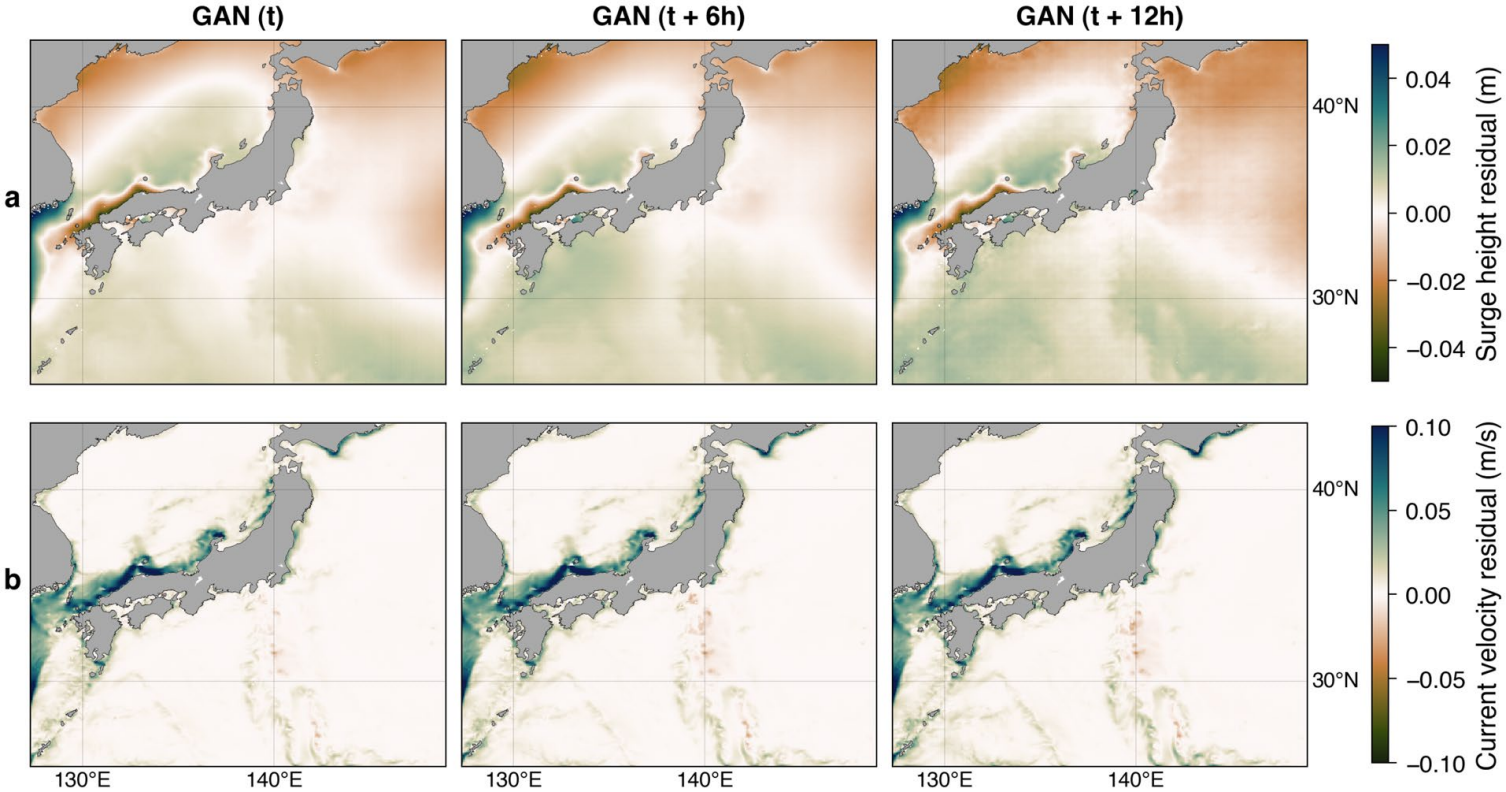
Maps of mean residual of surge height (**a**) and ocean current velocity (**b**). The residual (NWP–GAN) is averaged over the test set. The map was created using the Matplotlib Basemap Toolkit (https://matplotlib.org/basemap/) in Python.

To more clearly visualize the performance of the storm surge simulation using forcings from the GAN model relative to the NWP model, we plot the maps of mean residual in Fig. 9. The mean residual is defined by subtracting the GAN-based storm surge simulations from the NWP-based results averaged over the test set. The maps show the overestimation tendency of the surge height to the north of the modeling domain, where the extratropical transition commonly occurs (Fig. 9a). The overestimation is also apparent in the west coast of Japan collocated with the distinctly underestimated ocean current velocity (Fig. 9b) caused by the limitation of the GAN model in simulating the northwest quadrant of typhoons as explained above. These areas mark where the GAN-based storm surge simulation is less accurate. Conversely, the storm surge simulation with forcings from the GAN and NWP models exhibit comparable performance on the east coast, particularly around Tokyo Bay.

**Figure 10:**
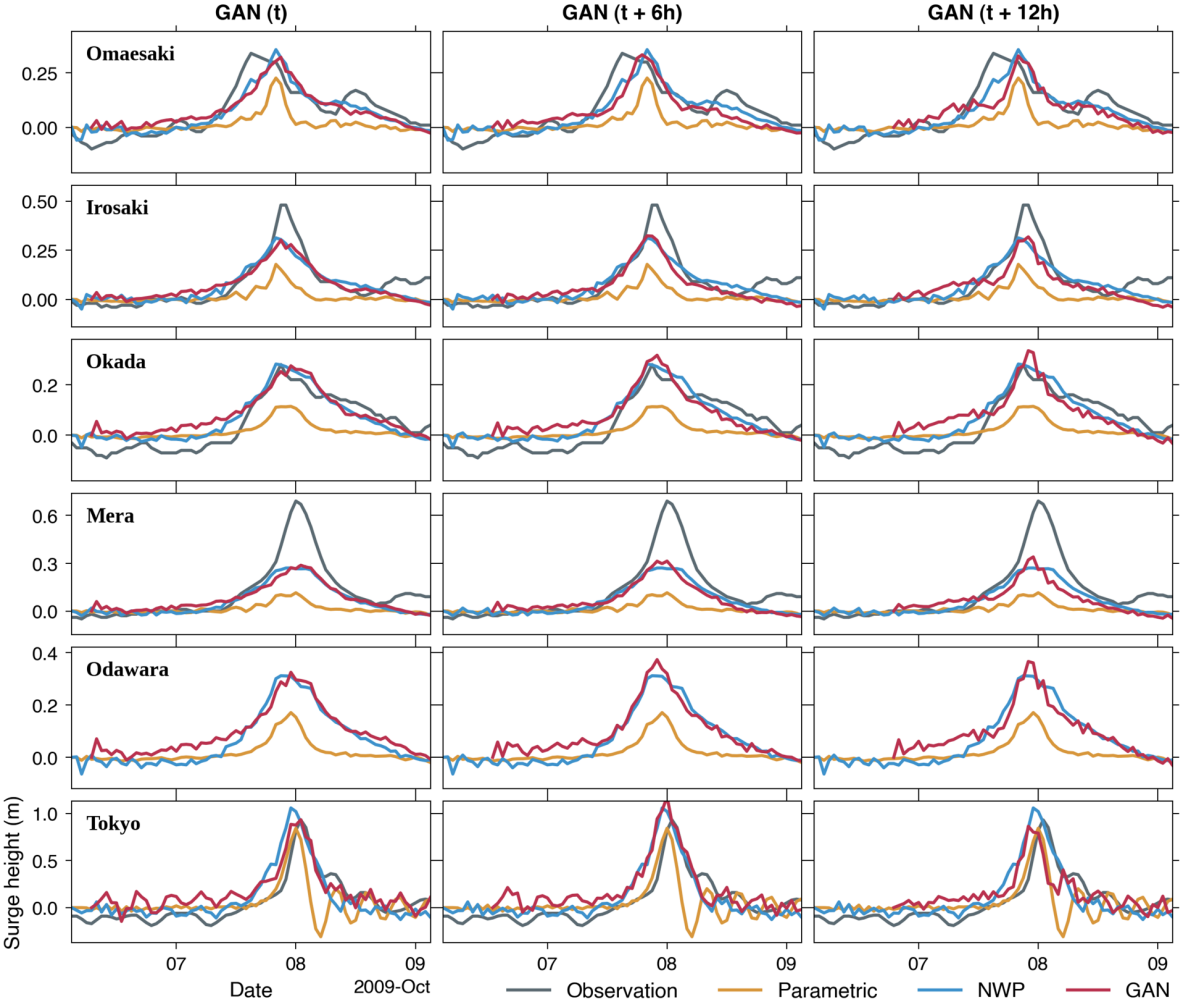
Comparisons between observed and simulated surge heights during the 2009 Typhoon Melor using forcings from the parametric model, the NWP model, and GAN models at *t*, *t* + 6 h, and *t* + 12 h. The observation at Odawara is unavailable for this event.

Comparisons of time series of surge levels at tidal stations further demonstrate the performance of our proposed GAN models (Fig. 10). At all stations, the predicted surge heights using forcing from GAN models with various lead times are in good agreement with those of the NWP model. This result indicates that the proposed GAN model can be a potential surrogate for the NWP model, which outperforms the parametric model. However, the simulated storm surges with all different forcings considerably underestimate the observed surge peaks of ~ 0.5 m at Irosaki and ~ 0.6 m at Mera. As the underestimation at Irosaki and Mera is repeated for other events (Supplementary Figs. 16–18), the possible cause is likely ascribed to the insufficient grid resolution or bathymetry data used in the hydrodynamics simulation. Local coastal effects have been reported to contribute to the total water level during typhoons^6,20^. Specifically, the wave setup at Mera is critical as it faces the open ocean, influencing the total water level. Thus, the underestimation seems to be natural since the storm surge model did not consider the wave effect.

### Computing time and storm surge model error

While the storm surge computational cost is the same for all the models, the main contribution of the proposed method is demonstrated in the computing time of the atmospheric forcings (Table 1). Since no information is available on the computational cost for the DSJRA dataset, we refer to another study with a nearly similar NWP model domain and horizontal grid size^48^. Although their NWP model may have different configurations to the DSJRA and computational resources to our study, the comparison provides an overview of the typical computational cost required by NWP models. We train the GAN models using an NVIDIA A100 (80 GB) graphical processing unit, by which the computing time for a typhoon event takes approximately 9 s. The fully trained GAN model is also run on a standard desktop computer with a single central processing unit to provide a fair comparison with the parametric model, as shown in Table 1. Table 1 also indicates the storm surge model error based on the RMSE relative to the observation at all considered tide gauges for the 2009 Typhoon Melor shown in Fig. 10.

**Table 1:** The computing time for generating atmospheric forcings and the corresponding storm surge model error.

Model		Computing time (s)	RMSE (m)
Parametric		8	0.16
‍NWP		4200*	0.10
‍‍GAN‍	t	13	0.10
	*t* + 6 h		0.12
	*t* + 12 h		0.13

We use a standard desktop computer with a single processor for the parametric and GAN models for a 73-h simulation time of the 2009 typhoon Melor.

*The computation was performed on the FUJITSU supercomputer FX100 with 256 processors for a 45-h integration time of a different event^48^.

### Uncertainty

**Figure 11:**
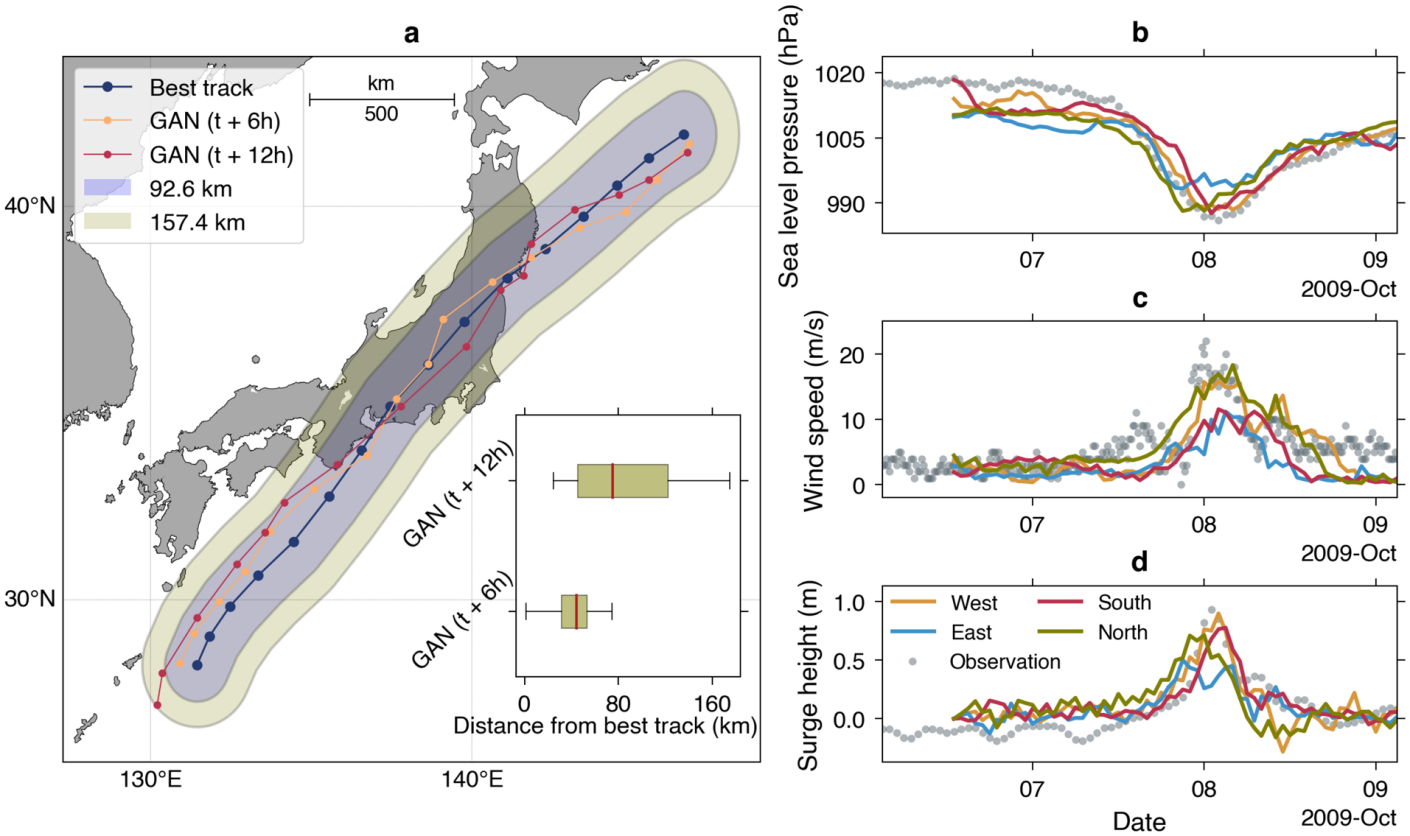
(**a**) Predicted tracks and the best track of the 2009 Typhoon Melor overlaid with the composites of probability-circle radii of 92.6 km and 157.4 km. The inset shows box plots of distances between the best and predicted tracks. Ensemble predictions at Tokyo station for the 6-h lead time of the sea level pressure (**b**), the wind speed (**c**), and the surge height (**d**). The map was created using the Matplotlib Basemap Toolkit (https://matplotlib.org/basemap/) in Python.

The main source of uncertainty in the storm surge modeling is strongly linked to the typhoon track^17,49^. To assess the uncertainty of our simulation results, we use the 2009 Typhoon Melor forecasts as an example. Figure 11a compares the best track and the tracks extracted from GAN models with the specified lead times. The medians of distances of the respective track relative to the best track are 44.2 km and 75.3 km for predictions at *t* + 6 h and *t* + 12 h, respectively (inset of Fig. 11a). Our result is comparable to a previous study emphasizing the typhoon track prediction from satellite images with an average error of 95.6 km for a 6-h lead time^50^. Furthermore, the track uncertainty is also appraised in the context of the probability-circle radii for typhoon track forecasts determined by the JMA^51^. The probability-circle radii were categorized into groups of different wind speeds for various forecasts or lead times. For convenience, here we only use the expected values for a wind speed of > 30 kt based on the evaluation done in 2016^51^. For 6-h and 12-h lead times, the expected probability-circle radii are 50 nm (= 92.6 km) and 85 nm (= 157.4 km), respectively. Figure 11a shows that the predicted tracks lie within the composite of the above radii. We acknowledge that the JMA is implementing a new categorization and verification, resulting in smaller probability-circle radii^52^. However, it does not alter the main conclusion of our study. Besides, we opt to use larger radii to more clearly visualize the effect of track deviations in our storm surge models.

The standard ensemble storm surge prediction is obtained by simulating the hydrodynamics using multiple predicted typhoon tracks as realized in the JMA forecasting system^49^. Here, we test a slightly different approach because the lead times have been defined beforehand under the GAN architecture. To create an ensemble storm surge prediction, we shift the input of the GAN models in four directions: west, east, south, and north following a previous study^17^. For conciseness, we only experiment using the 6-h lead time; thus, the amount of shifting corresponds to the 92.6 km of the JMA probability-circle radii. Figure 11b–d shows the predicted sea level pressure, wind speed, and the corresponding surge height at Tokyo station from all ensemble members. The time series in the forcings of the shifted cases varies with the difference in the wind speeds up to approximately 10 m/s. The simulated surge heights reflect the variability of the forcings and capture the observed maximum surge heights within the ensemble range. This experiment demonstrates the efficiency of GAN as the computational cost of this kind of prediction is similar to that of using the parametric model.

## Conclusions and future works

We have demonstrated the application of deep learning through our newly-proposed approach based on GAN to emulate the atmospheric forcing fields for simulating storm surges efficiently. The speed-accuracy tradeoff, which is considered the primary objective of this study, typically encountered in storm surge modeling, has been addressed. Furthermore, some of the physical properties associated with typhoons while undergoing an extratropical transition or landfall are reasonably attained, which was previously difficult to accomplish using conventional parametric models. The proposed method in this study can improve the standard numerical storm surge modeling with forcings from the parametric model and is also equipped with a useful forecasting feature with up to 12-h lead time. This feature, together with the cost-effectiveness of the algorithm, will be favorable for an operational storm surge forecasting system, especially when access to high-performance computing is unavailable or is limited. However, in the future, several potential improvements can be made to this method from both scientific and practical perspectives, as discussed below.

A control for the GAN model could be introduced likely through an attention mechanism^53^ to focus on the more informative components, which in our case is an area surrounding the typhoon center. It will contribute to the storm surge accuracy sensitive to the typhoon core region. Another way to attain such an improvement is to train the GAN model using a hybrid NWP and parametric model^54^. However, precautions should be taken concerning the neglected topographic effect in the parametric model part. Further improvements in the hydrodynamics section, apart from using higher resolution grid and bathymetric data, can be achieved by simulating the nonlinear interactions between tide, wave, and surge using coupled models^55^. The comprehensive modeling framework provides a more accurate physical representation of hydrodynamic responses to the atmospheric disturbance by a typhoon, albeit it requires extra computational efforts.

On the contrary, from the practical point of view, a more efficient storm surge numerical model^15^ is one alternative to realizing a rapid forecasting system. Additionally, configuring the GAN model to directly translate atmospheric forcings into sea surface elevation fields can substantially speed up the real-time computations of storm surges. Since the 12-h lead time forecasts in this study show reasonable accuracy, the model can be extended further to account for longer lead times which will be crucial in operational storm surge forecasting. One may use a coarser time interval for the GAN model with longer lead times to preserve computer memory usage, particularly during training. Lastly, owing to the flexibility of the proposed method in fusing various datasets, auxiliary inputs from other related variables or satellite images may also be advantageous for prospective studies.

## Supplementary Information

Supplementary Information 1.

Supplementary Video 1.

Supplementary Video 2.

## Data Availability

The typhoon best track data of IBTrACS is available at https://www.ncei.noaa.gov/products/international-best-track-archive, and the reanalysis products of the DSJRA-55 are downloaded from https://search.diasjp.net/en/dataset/DSJRA55. The observed wind and sea level pressure of the JODC are obtained from https://www.jodc.go.jp/jodcweb/JDOSS/index.html, while the observed storm surge by the JMA can be found at https://www.data.jma.go.jp/gmd/kaiyou/db/tide/genbo/index.php. The bathymetry data are acquired from https://www.gebco.net/ and https://www.jha.or.jp/en/jha/.

## Code availability

The GAN (pix2pix) model is based on the Keras library in Python adopted from https://machinelearningmastery.com/how-to-develop-a-pix2pix-gan-for-image-to-image-translation/. The ROMS source code is available at https://www.myroms.org/. The parametric model is modified from https://github.com/ec-jrc/pyStorms. All figures are produced using the Matplotlib library implemented in Python (https://matplotlib.org/).
