# Supplementary material for: A novel deep learning approach for typhoon-induced storm surge modeling through efficient emulation of wind and pressure fields
Source: Sci Rep. 2023 May 16;13:7918. doi: 10.1038/s41598-023-35093-9 (PMC10188603; doi:10.1038/s41598-023-35093-9)
Supplement: Supplementary file 1 — Supplementary Information 1. [file 41598_2023_35093_MOESM1_ESM.docx]

Supplementary Information for

# **A novel deep learning approach for typhoon-induced storm surge modeling through efficient emulation of wind and pressure fields**

Iyan E. Mulia^1, 2*^, Naonori Ueda^1, 2^, Takemasa Miyoshi^1, 3^, Takumu Iwamoto^4^, Mohammad Heidarzadeh^5^

^1^Prediction Science Laboratory, RIKEN Cluster for Pioneering Research, Kobe, Japan.

^2^Disaster Resilience Science Team, RIKEN Center for Advanced Intelligence Project, Tokyo, Japan.

^3^Data Assimilation Research Team, RIKEN Center for Computational Science, Kobe, Japan.

^4^Tsunami and Storm Surge Research Group, Port and Airport Research Institute, Yokosuka, Japan.

^5^Department of Architecture and Civil Engineering, University of Bath, Bath , UK.

^*^iyan.mulia@riken.jp

Supplementary materials including:

**Supplementary Videos 1-2.**

**Supplementary Table 1.**

**Supplementary Figures 1-18.**

**Supplementary Video 1. S**imulated sea level pressure and wind fields of the 2009 Typhoon Melor by the parametric model, the NWP model, and GAN models at *t*, *t*+6h, and *t*+12h.

**Supplementary Video 2. S**imulated storm surge and depth-averaged ocean currents during the 2009 Typhoon Melor using forcings from the parametric model, the NWP model , and GAN models at *t* , *t*+6h, and *t*+12h.

**Supplementary Table 1.** List of typhoon events and simulation times. Shaded rows mark the typhoon events used as test set.

| **Year** | **Name** | **Start** | **End** | **Hour** |
| --- | --- | --- | --- | --- |
| 1981 | Thad | 1981-08-18 15:00:00 | 1981-08-23 09:00:00 | 115 |
| 1981 | Gay | 1981-10-20 15:00:00 | 1981-10-23 03:00:00 | 61 |
| 1982 | Judy | 1982-09-09 12:00:00 | 1982-09-13 09:00:00 | 94 |
| 1983 | Abby | 1983-08-12 09:00:00 | 1983-08-21 09:00:00 | 217 |
| 1985 | Irma | 1985-06-28 21:00:00 | 1985-07-01 18:00:00 | 70 |
| 1985 | Ruby | 1985-08-27 18:00:00 | 1985-08-31 21:00:00 | 100 |
| 1986 | Roger | 1986-07-14 18:00:00 | 1986-07-18 12:00:00 | 91 |
| 1989 | Mac | 1989-08-02 06:00:00 | 1989-08-08 03:00:00 | 142 |
| 1989 | Wayne | 1989-09-17 03:00:00 | 1989-09-20 06:00:00 | 76 |
| 1990 | Winona | 1990-08-04 00:00:00 | 1990-08-11 09:00:00 | 178 |
| 1990 | Gene | 1990-09-26 03:00:00 | 1990-10-01 18:00:00 | 136 |
| 1990 | Hattie | 1990-10-04 09:00:00 | 1990-10-08 18:00:00 | 106 |
| 1991 | Harry | 1991-08-28 03:00:00 | 1991-08-31 18:00:00 | 88 |
| 1991 | Luke | 1991-09-17 06:00:00 | 1991-09-20 00:00:00 | 67 |
| 1992 | Bobbie | 1992-06-27 21:00:00 | 1992-07-01 09:00:00 | 85 |
| 1993 | Vernon | 1993-08-23 21:00:00 | 1993-08-28 09:00:00 | 109 |
| 1996 | Violet | 1996-09-18 03:00:00 | 1996-09-23 15:00:00 | 133 |
| 1997 | Opal | 1997-06-18 09:00:00 | 1997-06-21 03:00:00 | 67 |
| 1997 | Peter | 1997-06-26 12:00:00 | 1997-06-29 06:00:00 | 67 |
| 1998 | Stella | 1998-09-13 21:00:00 | 1998-09-16 18:00:00 | 70 |
| 2000 | Kirogi | 2000-07-05 18:00:00 | 2000-07-09 06:00:00 | 85 |
| 2001 | Pabuk | 2001-08-18 03:00:00 | 2001-08-23 00:00:00 | 118 |
| 2001 | Danas | 2001-09-06 15:00:00 | 2001-09-12 12:00:00 | 142 |
| 2002 | Chataan | 2002-07-08 09:00:00 | 2002-07-12 00:00:00 | 88 |
| 2002 | Halong | 2002-07-13 21:00:00 | 2002-07-16 18:00:00 | 70 |
| 2002 | Higos | 2002-09-30 03:00:00 | 2002-10-02 03:00:00 | 49 |
| 2004 | Ma-on | 2004-10-07 15:00:00 | 2004-10-09 21:00:00 | 55 |
| 2004 | Tokage | 2004-10-18 12:00:00 | 2004-10-21 06:00:00 | 67 |
| 2005 | Banyan | 2005-07-24 06:00:00 | 2005-07-27 21:00:00 | 88 |
| 2005 | Mawar | 2005-08-21 18:00:00 | 2005-08-27 06:00:00 | 133 |
| 2006 | Maria | 2006-08-04 12:00:00 | 2006-08-11 12:00:00 | 169 |
| 2007 | Man-yi | 2007-07-12 12:00:00 | 2007-07-16 09:00:00 | 94 |
| 2007 | Fitow | 2007-09-02 00:00:00 | 2007-09-08 00:00:00 | 145 |
| 2009 | Krovanh | 2009-08-28 09:00:00 | 2009-09-01 15:00:00 | 103 |
| 2009 | Melor | 2009-10-06 03:00:00 | 2009-10-09 03:00:00 | 73 |
| 2011 | Roke | 2011-09-13 12:00:00 | 2011-09-22 06:00:00 | 211 |
| 2012 | Guchol | 2012-06-18 03:00:00 | 2012-06-22 06:00:00 | 100 |
| 2012 | Jelawat | 2012-09-28 06:00:00 | 2012-10-01 06:00:00 | 73 |


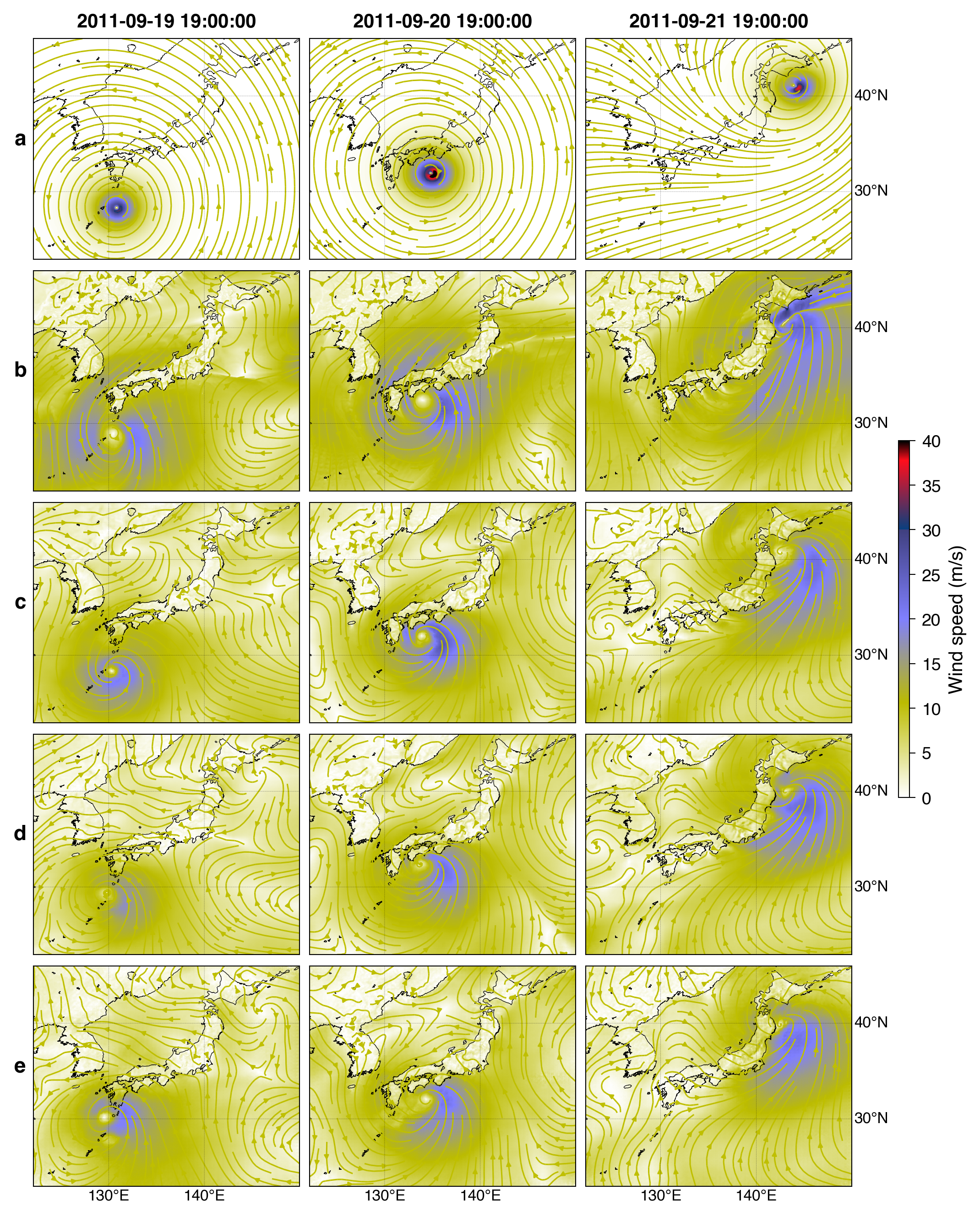
Supplementary Fig. 1. Snapshots of simulated wind fields of the 2011 Typhoon Roke using the parametric model (a), the NWP model (b), and GAN models at *t* (c), *t*+6h (d), and *t*+12h (e).


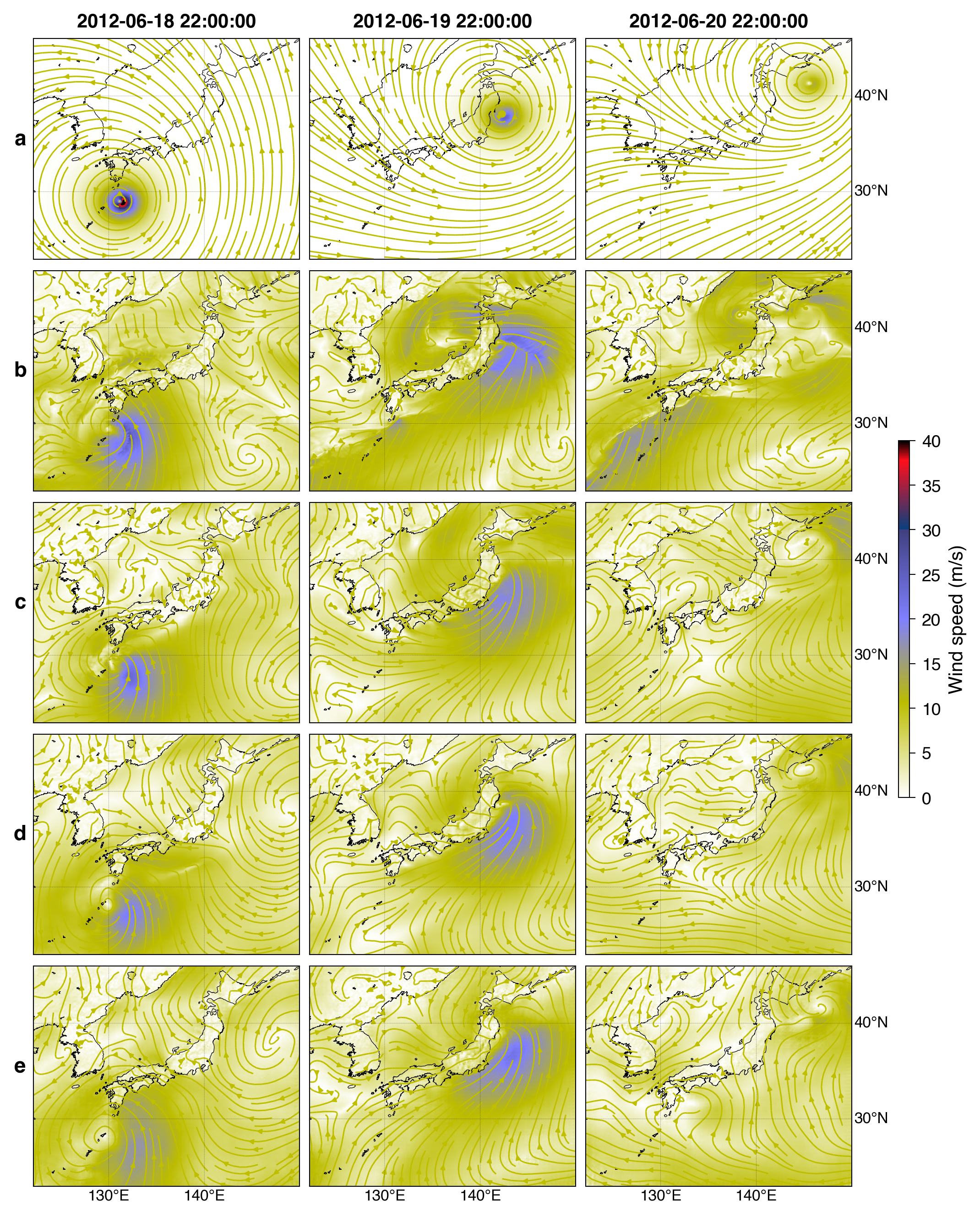
Supplementary Fig. 2. Same with Supplementary Fig. 1 for the 2012 Typhoon Guchol.


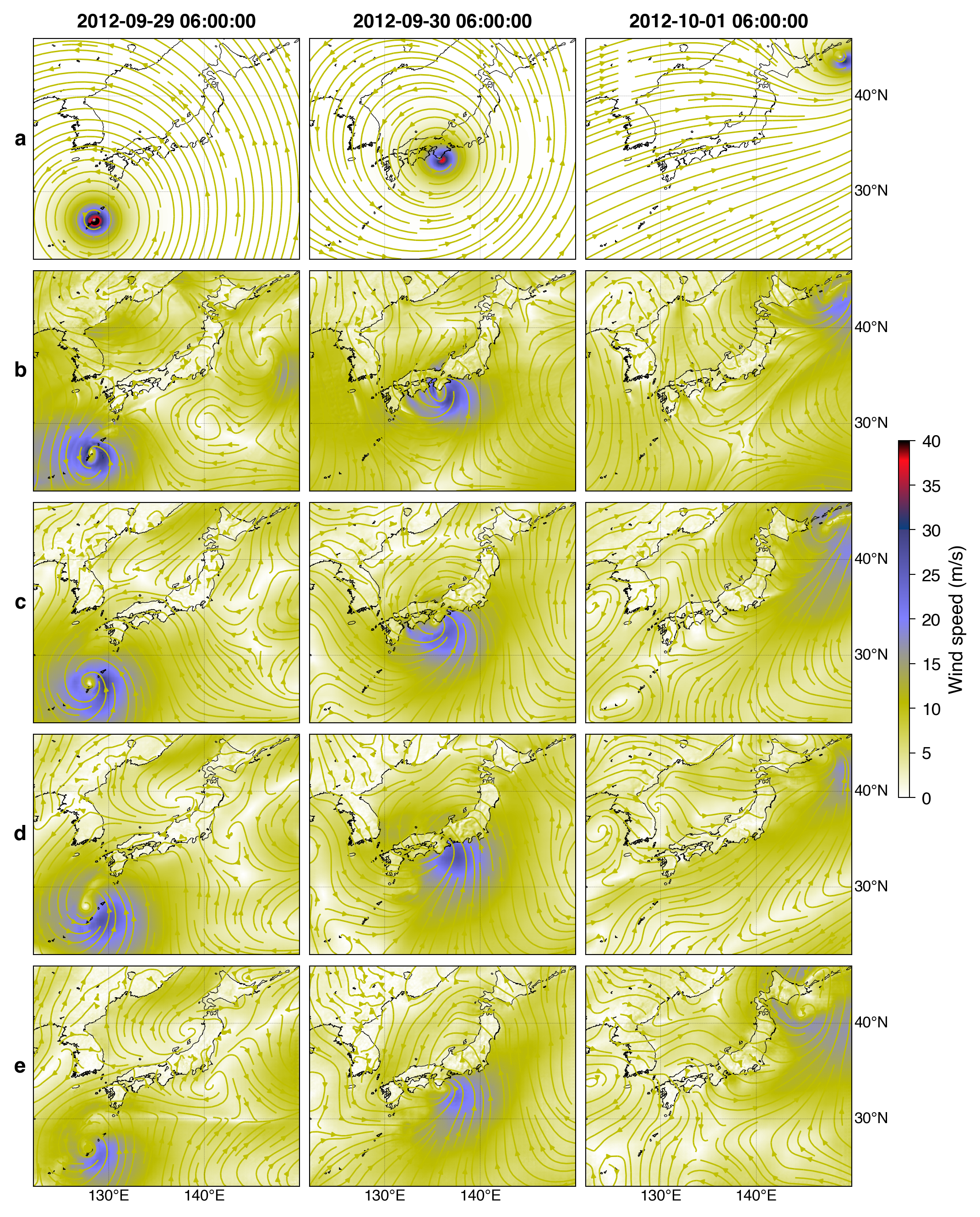
**Supplementary Fig. 3.** Same with Supplementary Fig. 1 for the 2012 Typhoon Jelawat.


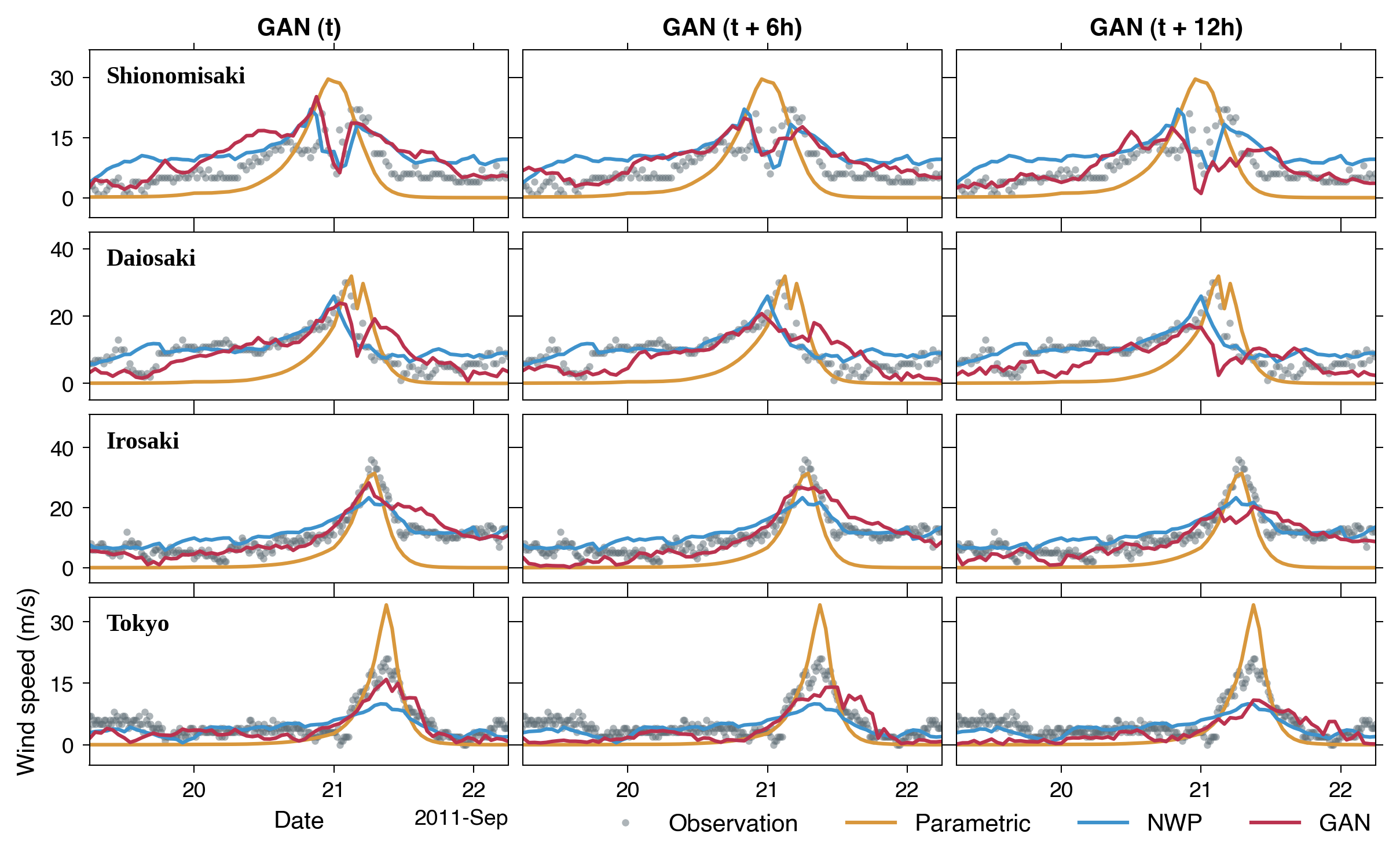
Supplementary Fig. 4. Comparisons between observed and simulated wind speed of the 2011 Typhoon Roke by the parametric model, the NWP model, and GAN models at *t*, *t*+6h, and *t*+12h.


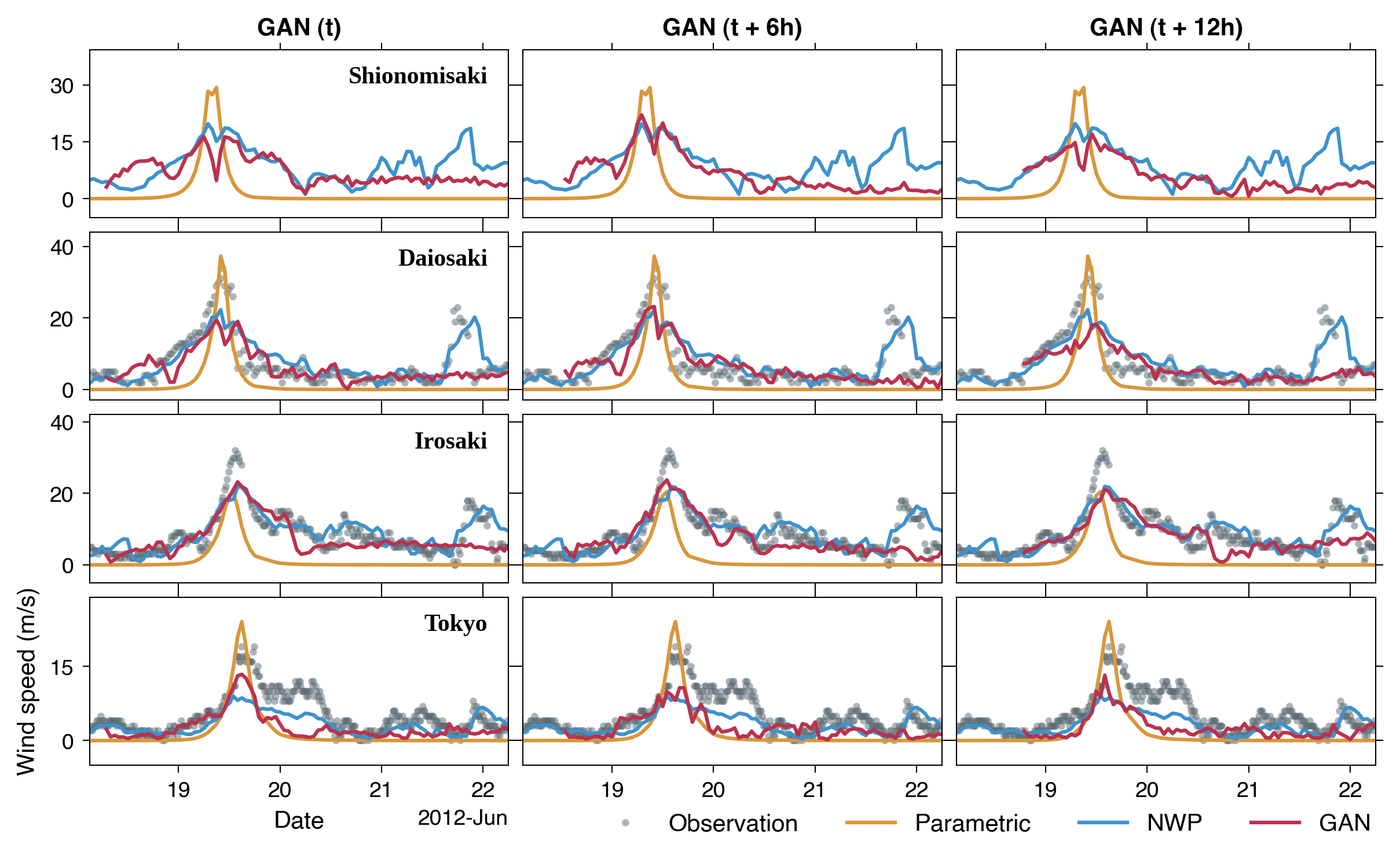
**Supplementary Fig. 5.** Same with Supplementary Fig. 4 for the 2012 Typhoon Guchol. The observation at Shionomisaki is unavailable for this event.


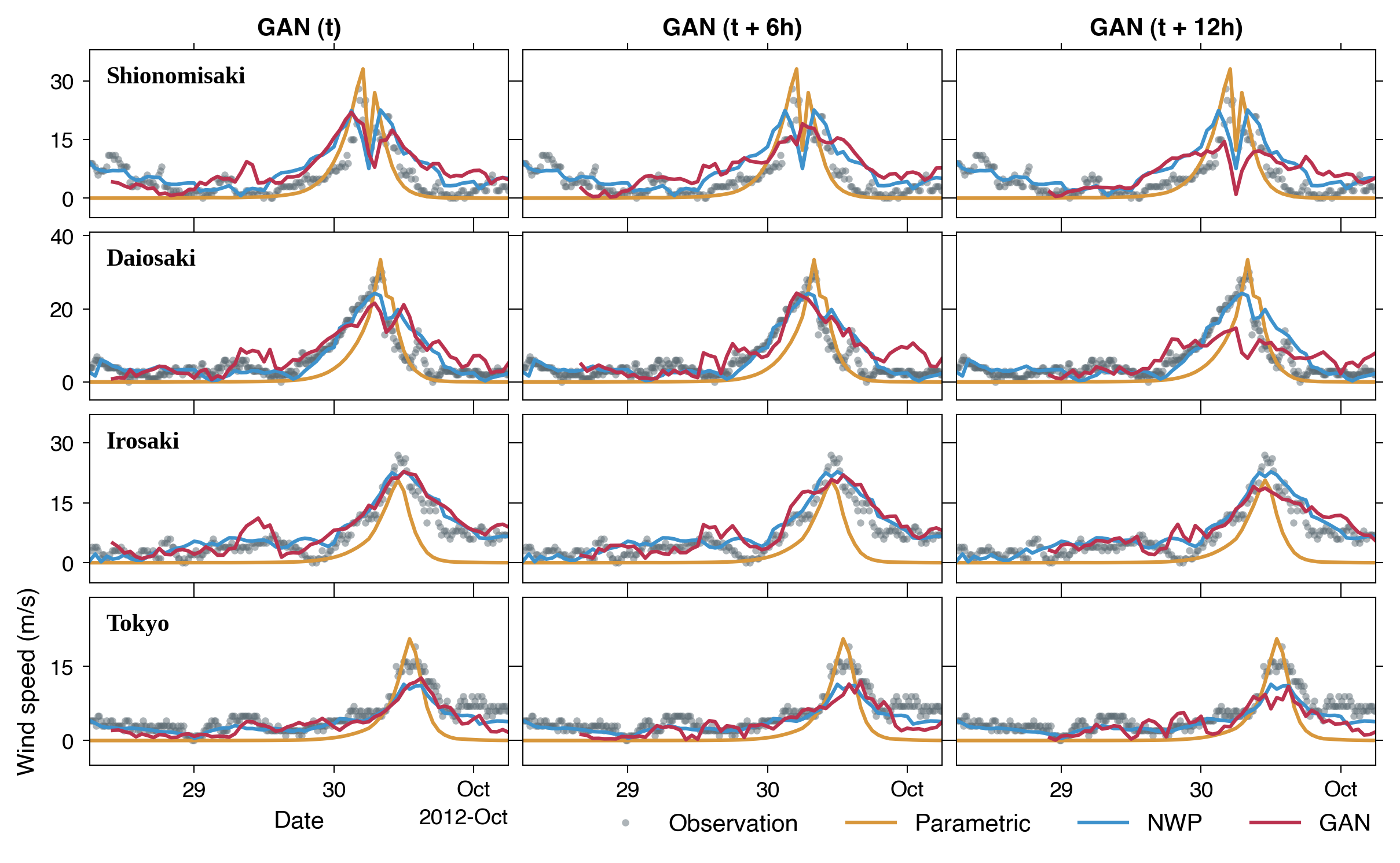
**Supplementary Fig. 6.** Same with Supplementary Fig. 4 for the 2012 Typhoon Jelawat.


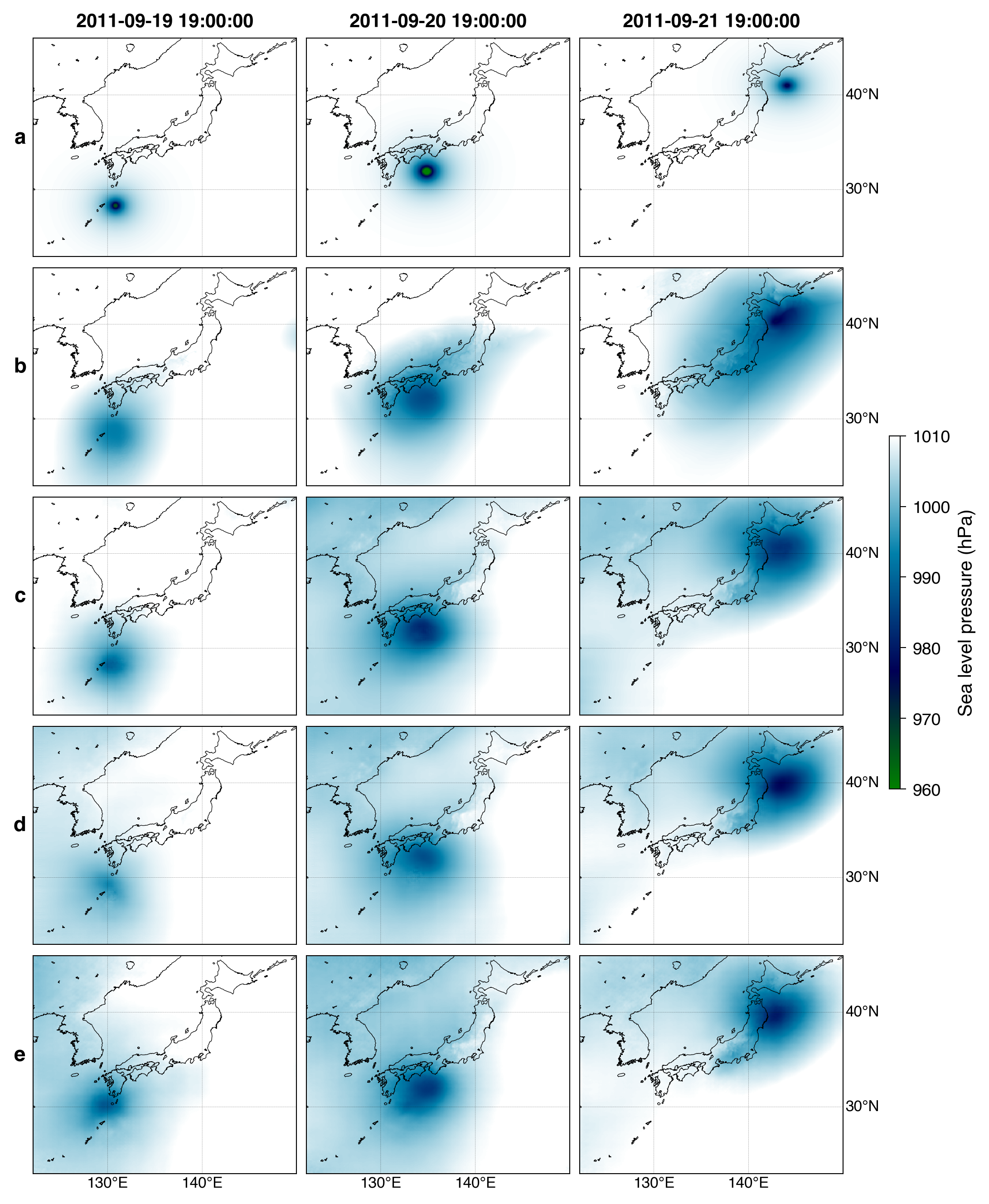
**Supplementary Fig. 7.** Snapshots of simulated sea level pressure fields of the 2011 Typhoon Roke using the parametric model (a), the NWP model (b), and GAN models at *t* (c), *t*+6h (d), and *t*+12h (e).


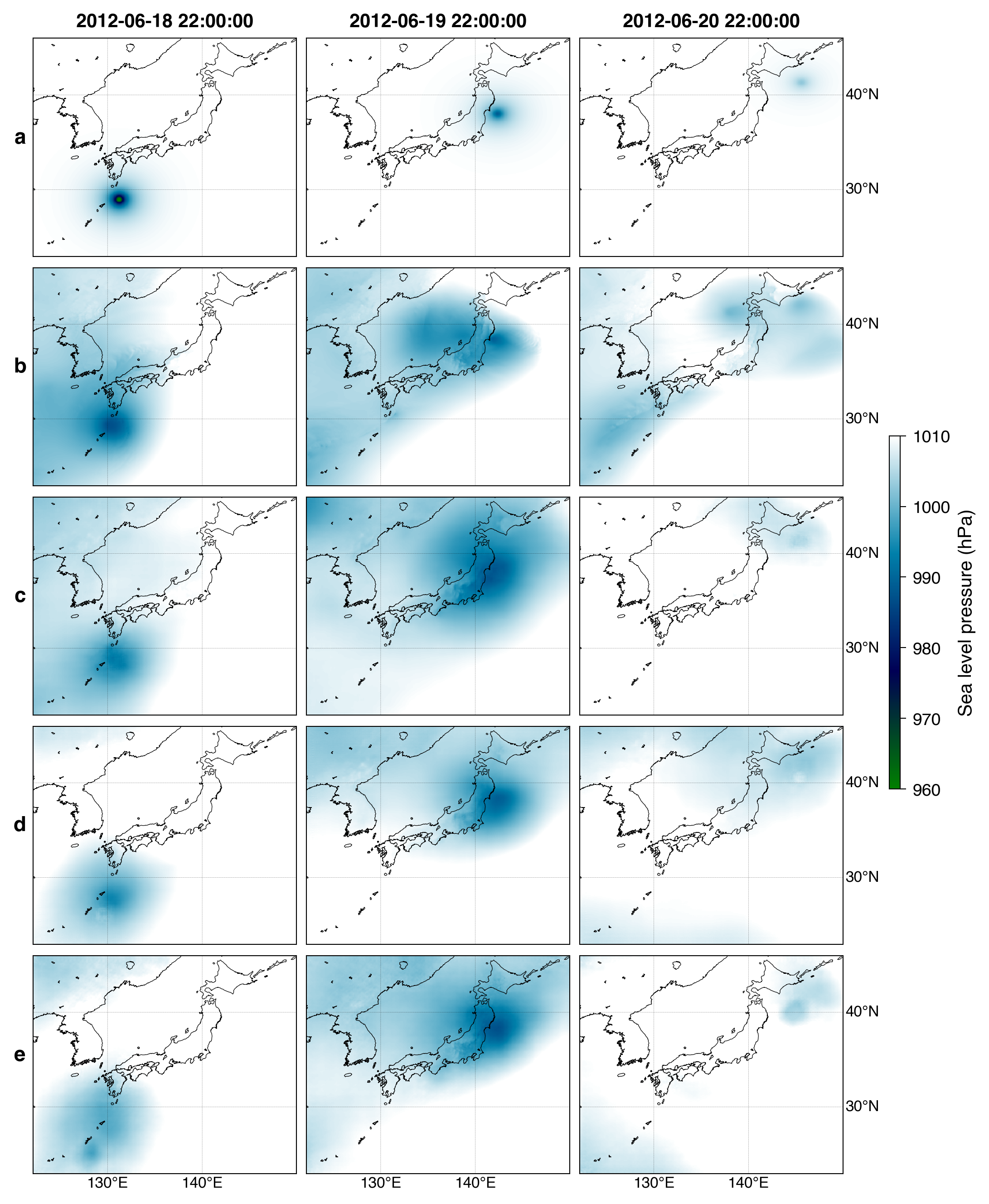
**Supplementary Fig. 8.** Same with Supplementary Fig. 7 for the 2012 Typhoon Guchol.


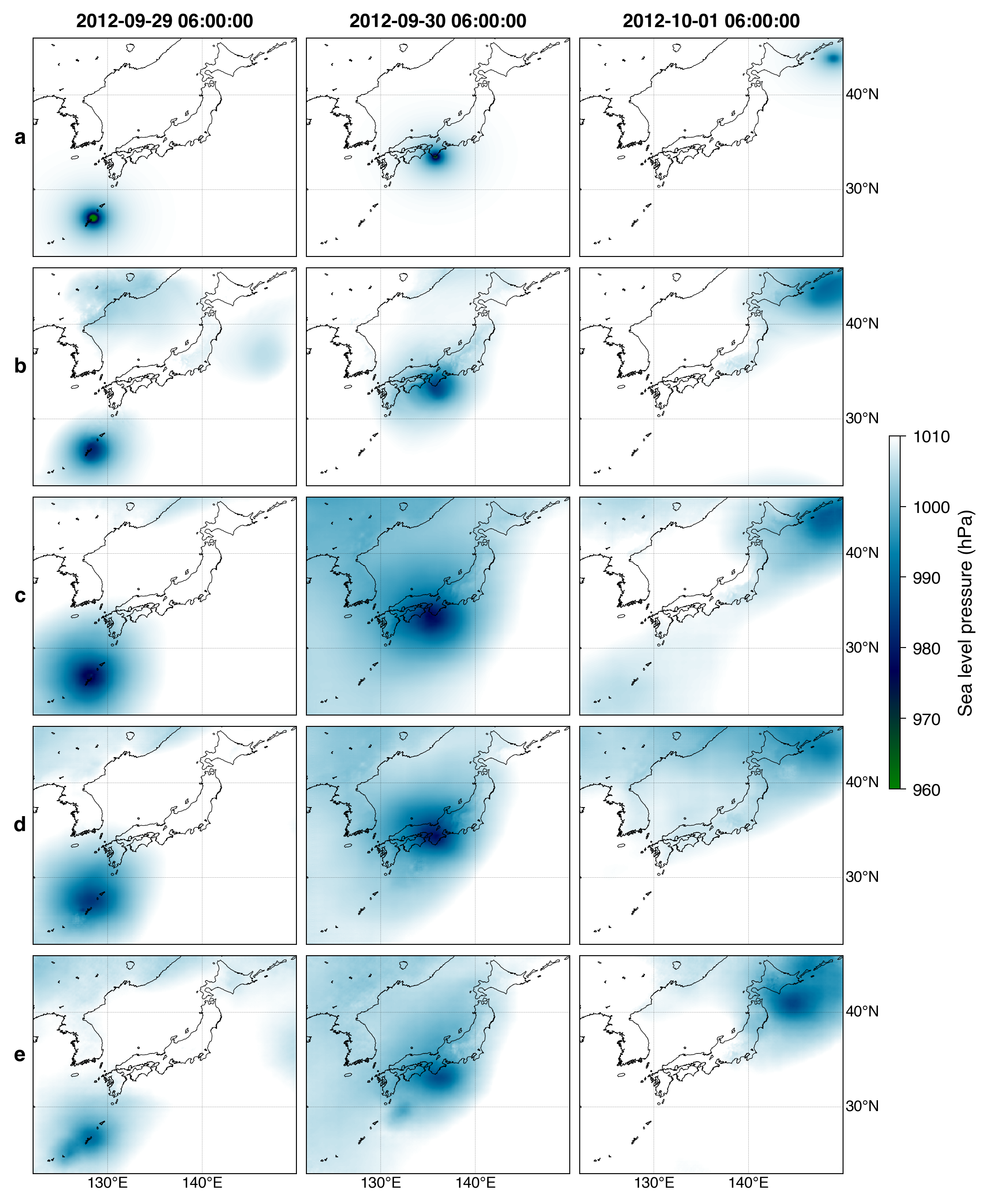
**Supplementary Fig. 9.** Same with Supplementary Fig. 7 for the 2012 Typhoon Jelawat.


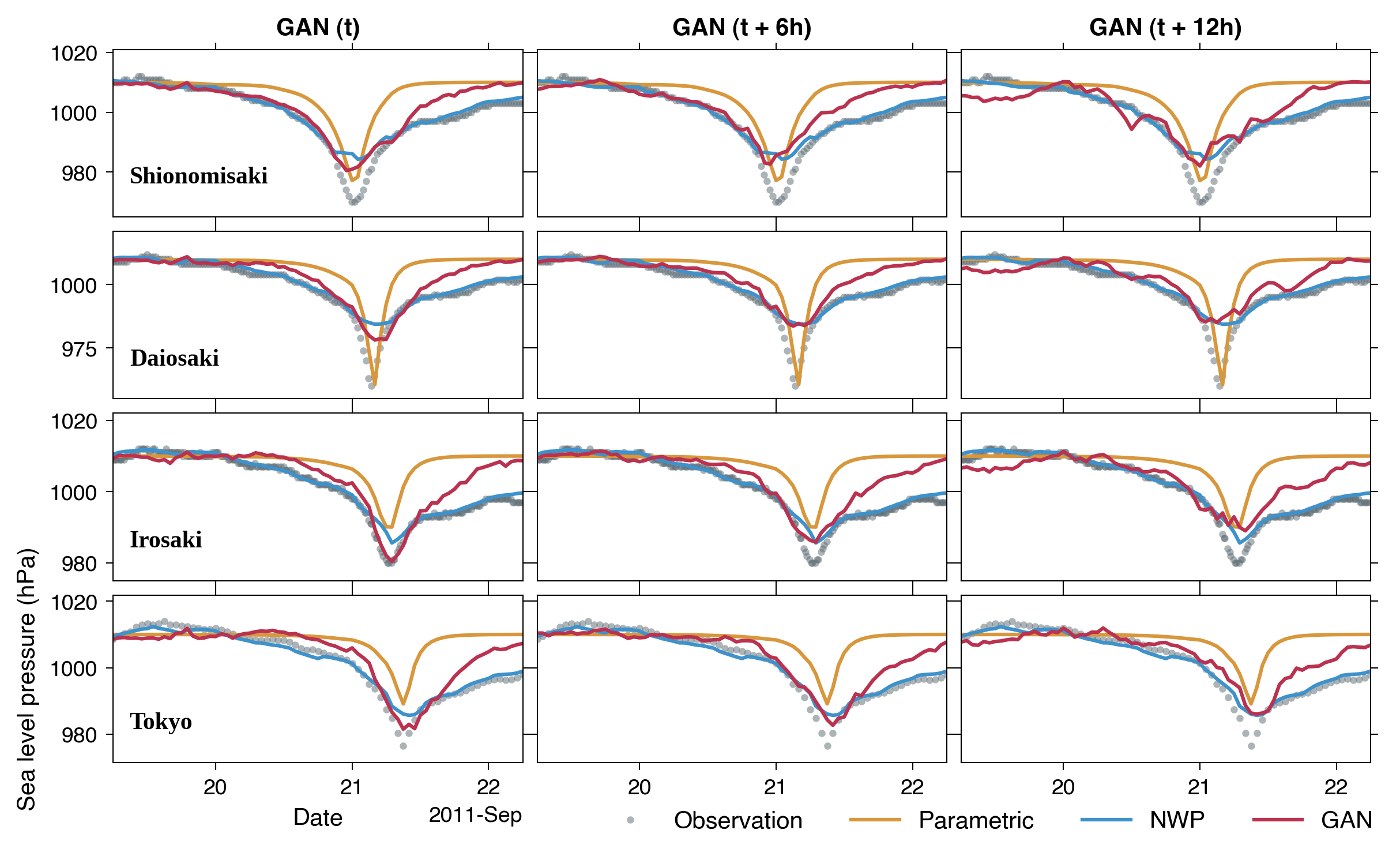
**Supplementary Fig. 10.** Comparisons between observed and simulated sea level pressure of the 2011 Typhoon Roke by the parametric model, the NWP model, and GAN models at *t*, *t*+6h, and *t*+12h.


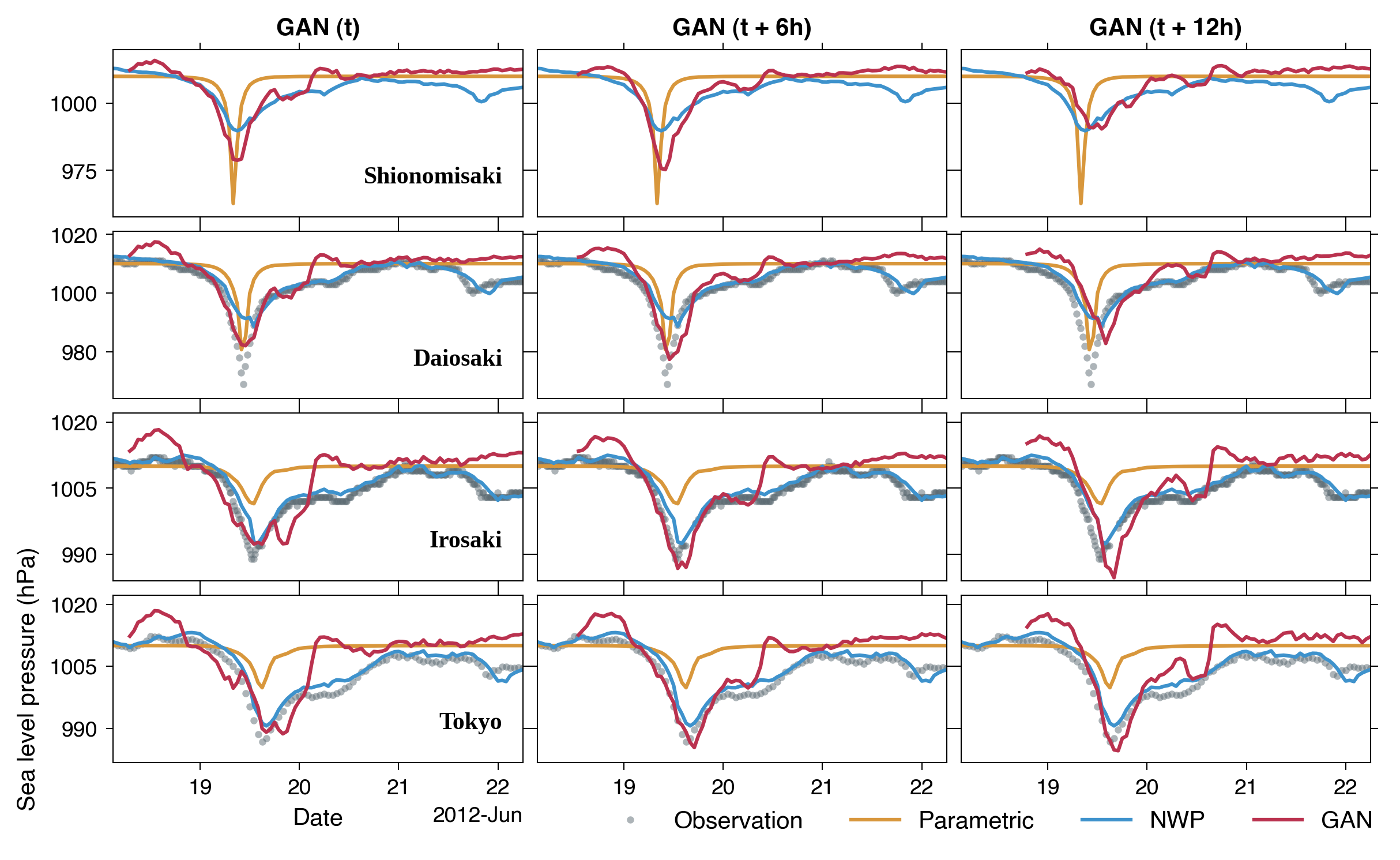
**Supplementary Fig. 11.** Same with Supplementary Fig. 10 for the 2012 Typhoon Guchol. The observation at Shionomisaki is unavailable for this event.


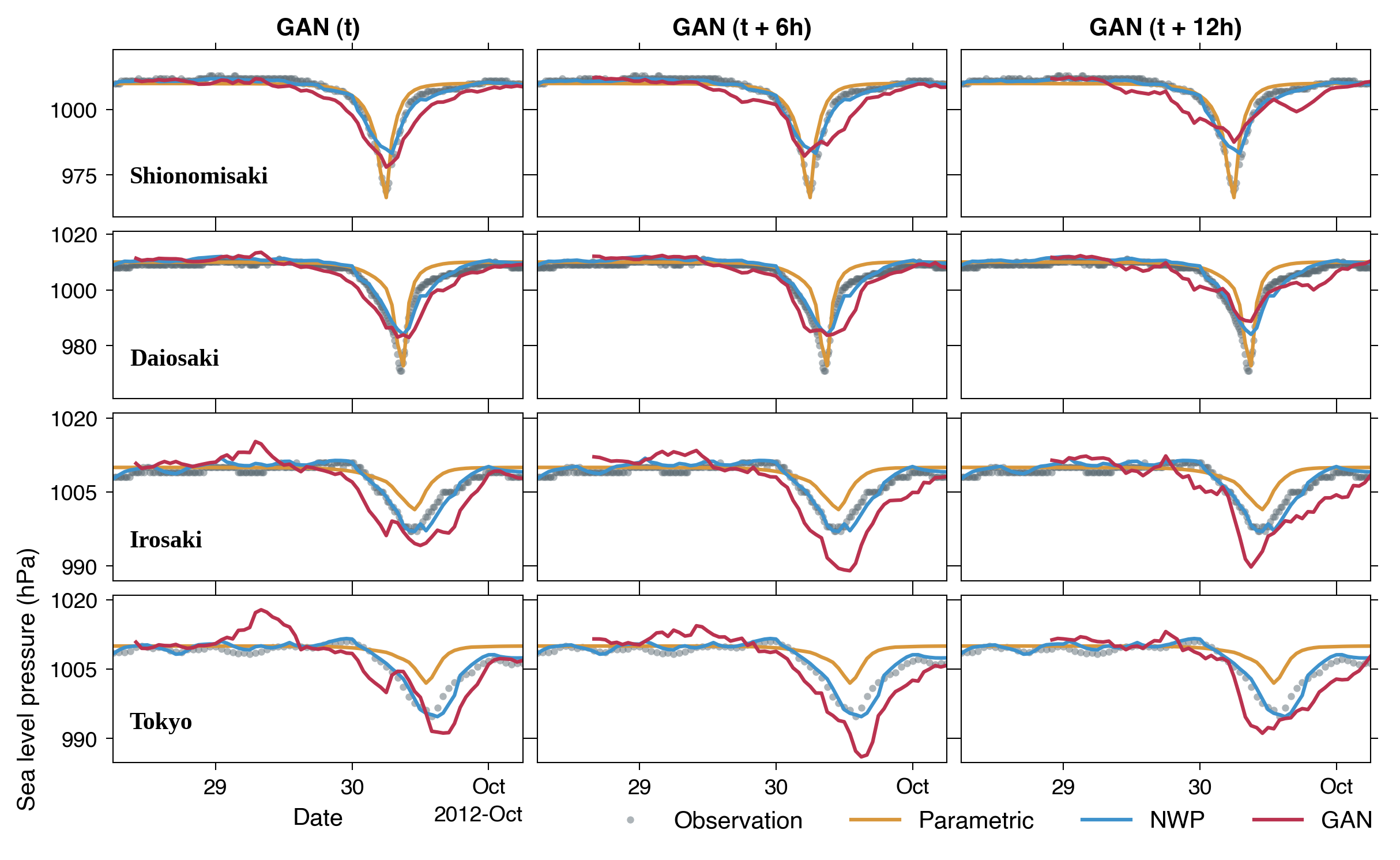
 **Supplementary Fig. 12.** Same with Supplementary Fig. 10 for the 2012 Typhoon Jelawat.


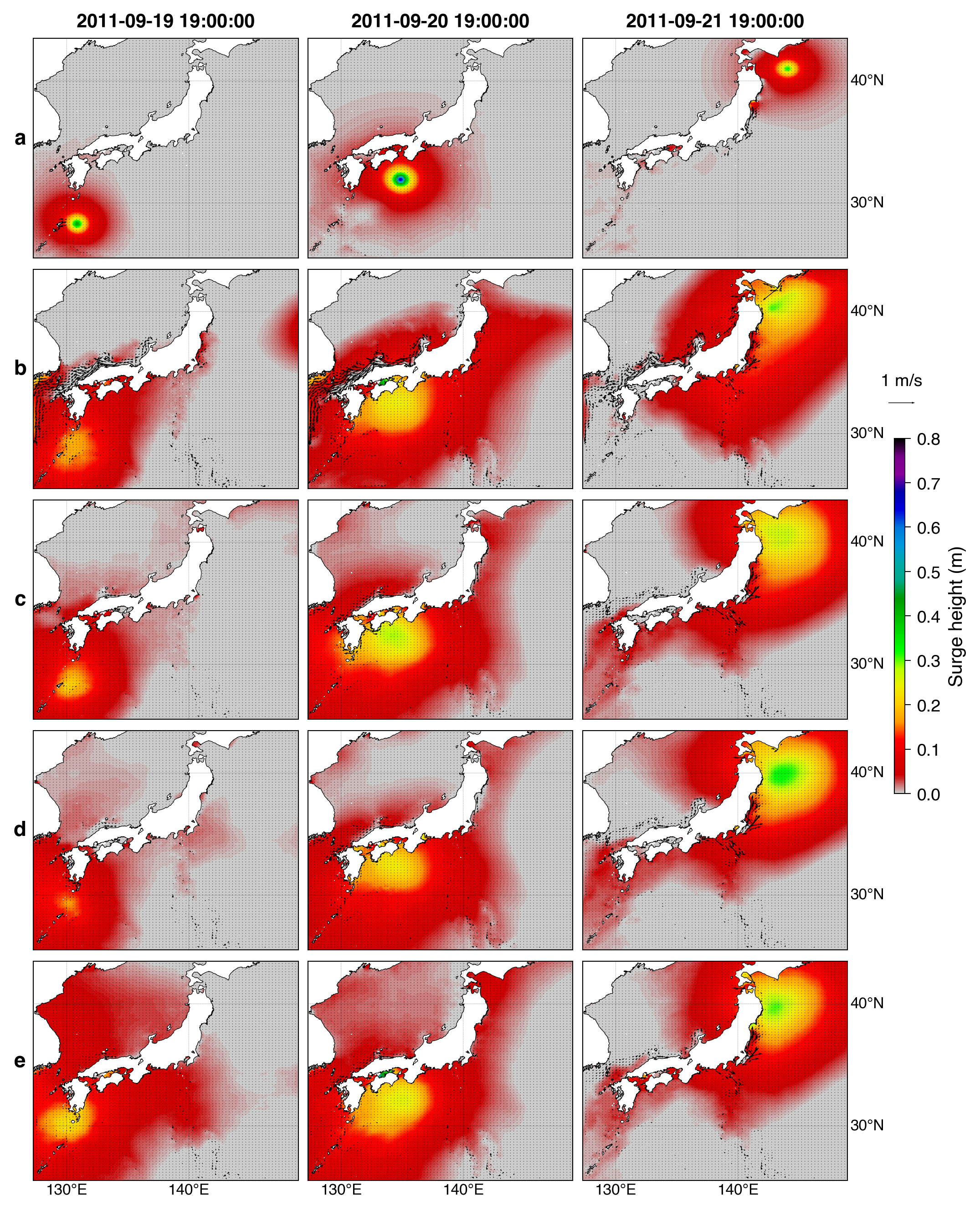
 **Supplementary Fig. 13.** Snapshots of simulated storm surge during the 2011 Typhoon Roke using forcings from the parametric model (a), the NWP model (b), and GAN models at *t* (c), *t*+6h (d), and *t*+12h (e).


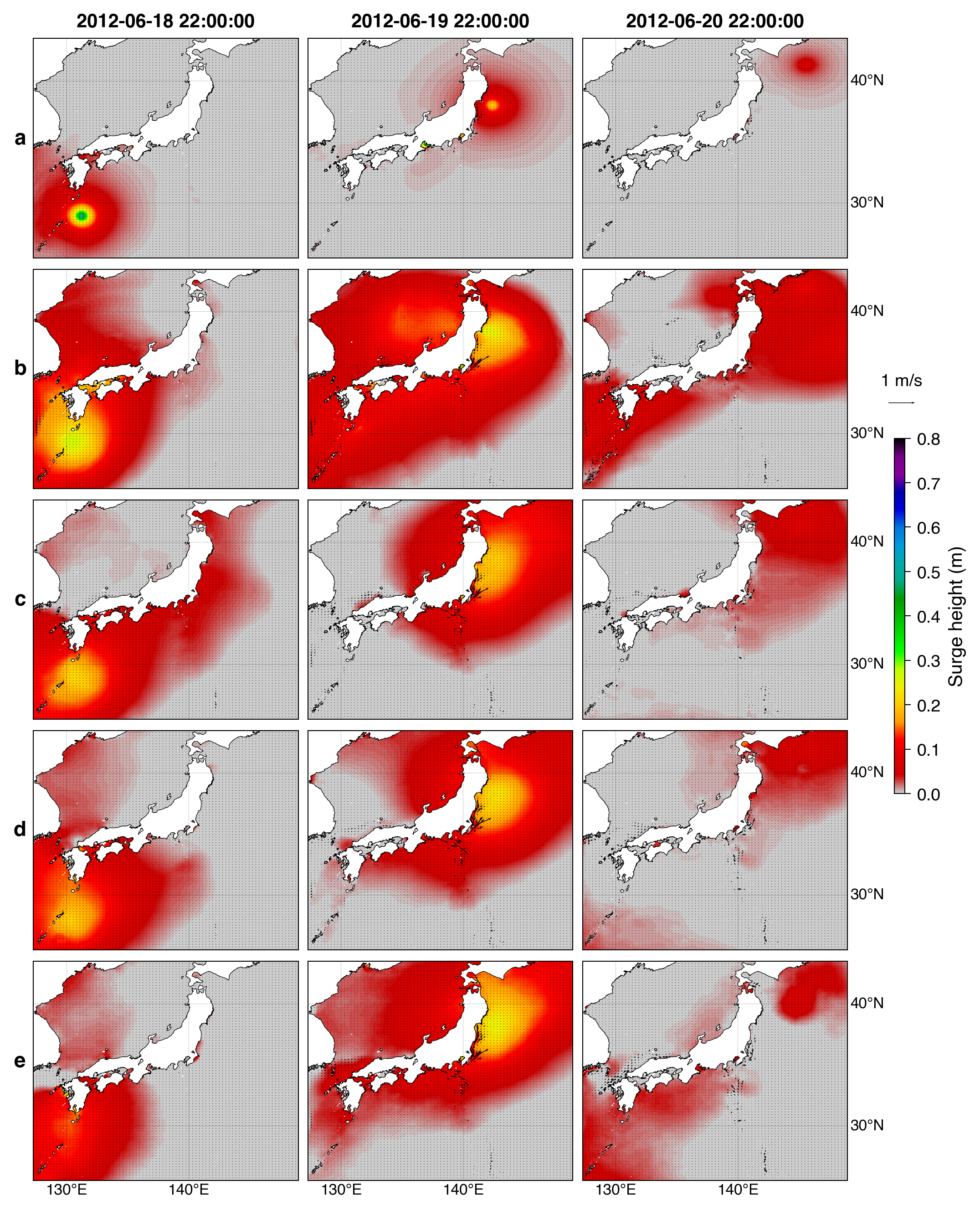
**Supplementary Fig. 14.** Same with Supplementary Fig. 13 for the 2012 Typhoon Guchol.


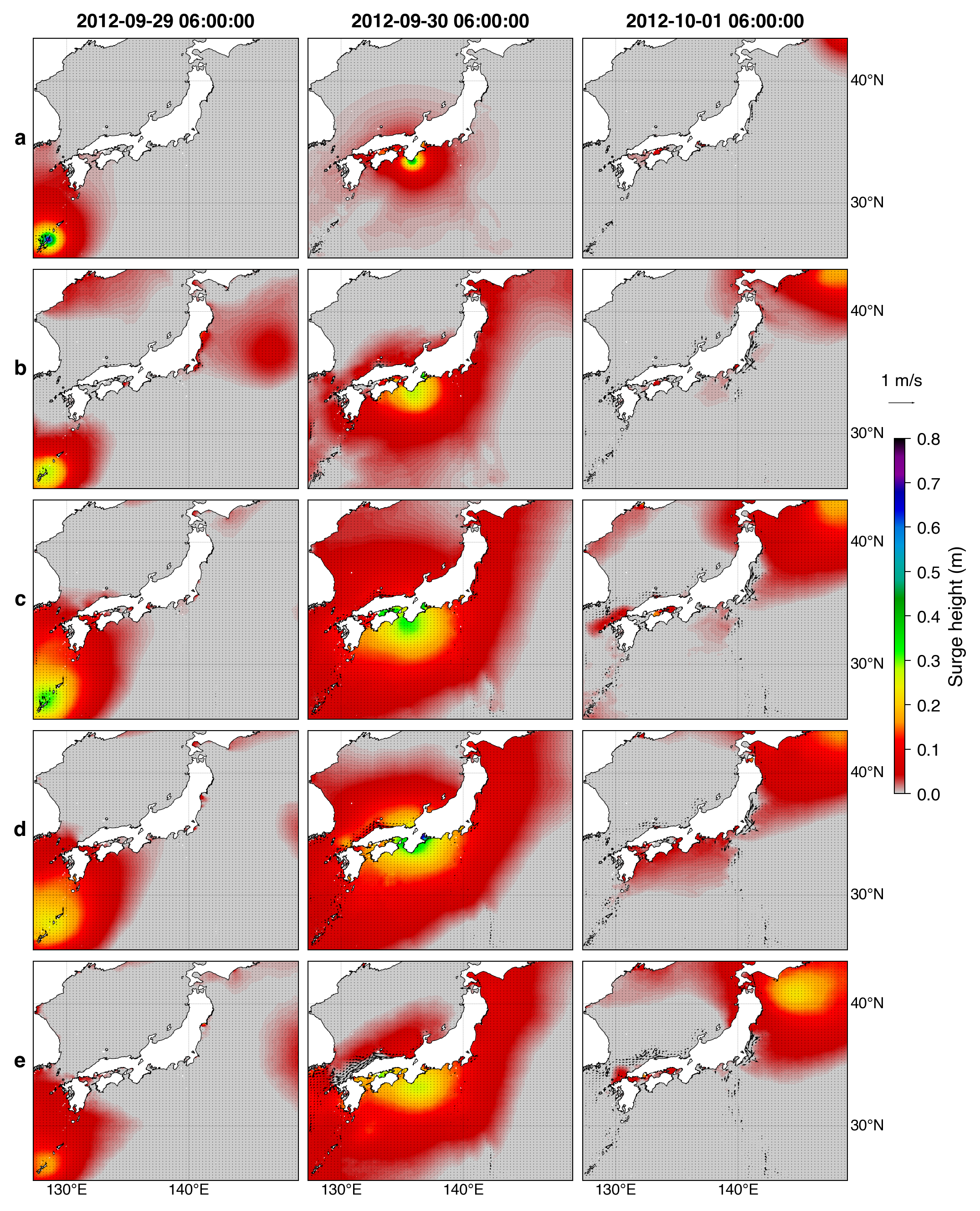
**Supplementary Fig. 15.** Same with Supplementary Fig. 13 for the 2012 Typhoon Jelawat.


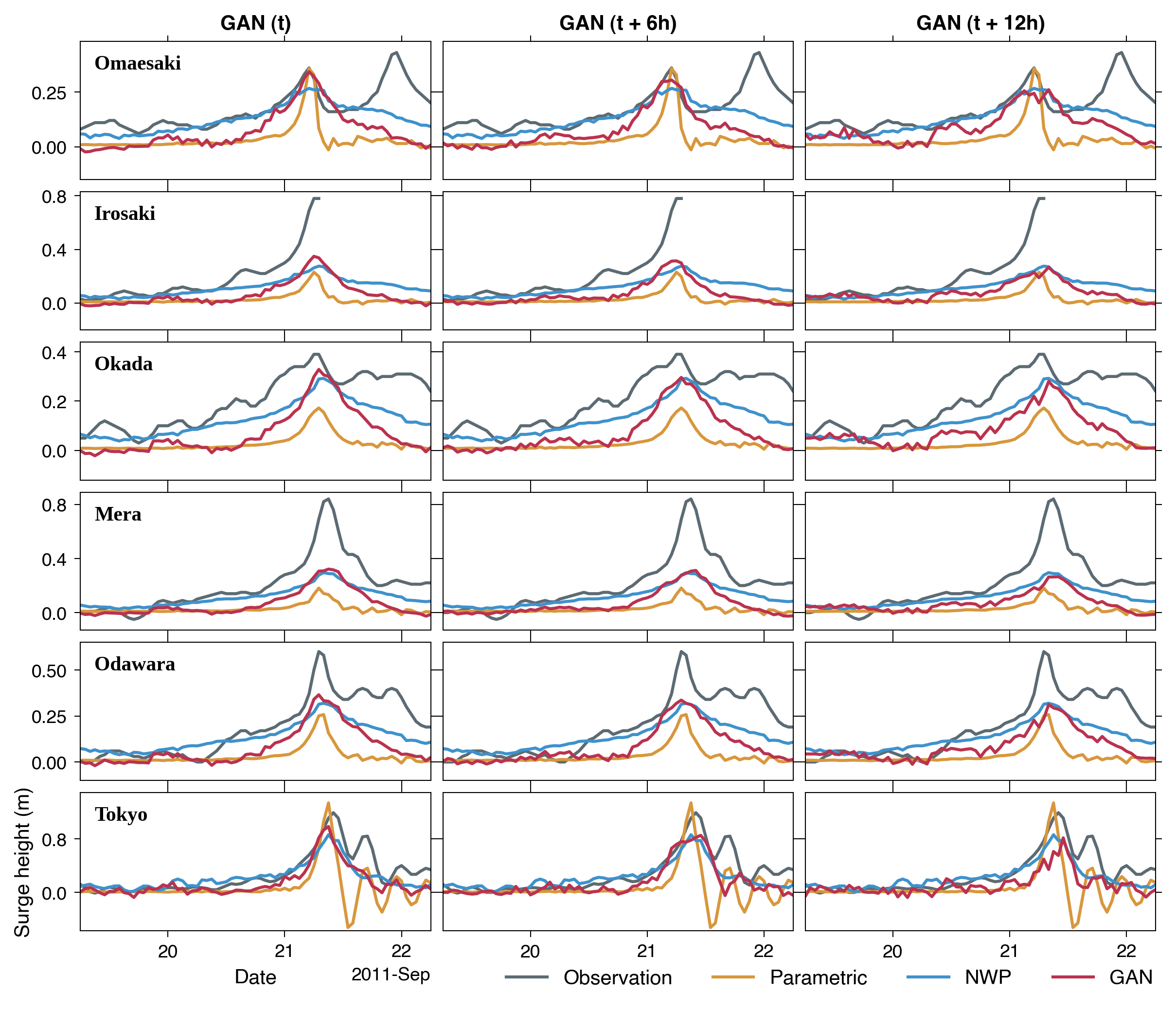
**Supplementary Fig. 16.** Comparisons between observed and simulated surge heights during the 2011 Typhoon Roke using forcings from the parametric model, the NWP model, and GAN models at *t*, *t*+6h, and *t*+12h.


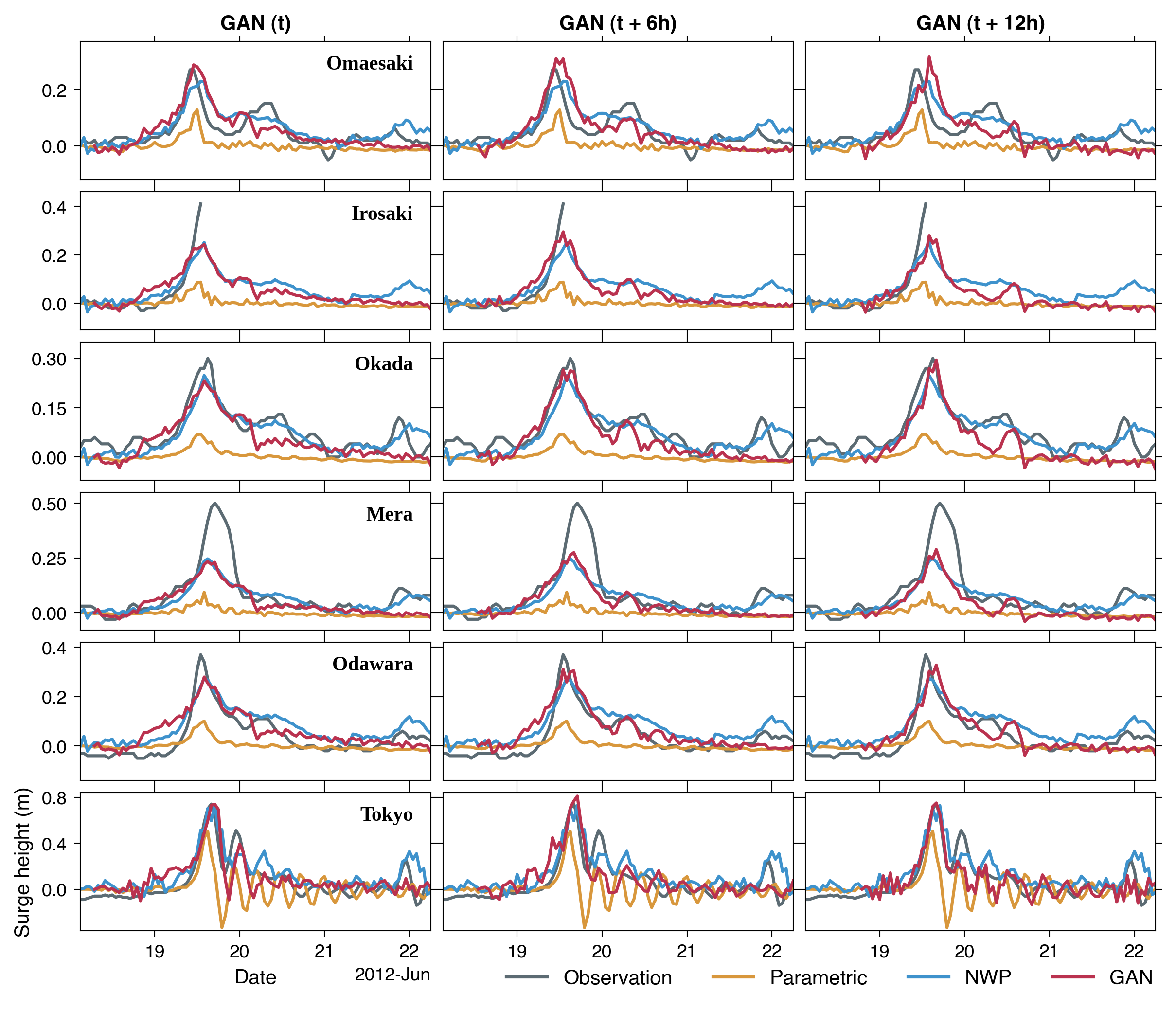
**Supplementary Fig. 17.** Same with Supplementary Fig. 16 for the 2012 Typhoon Guchol.


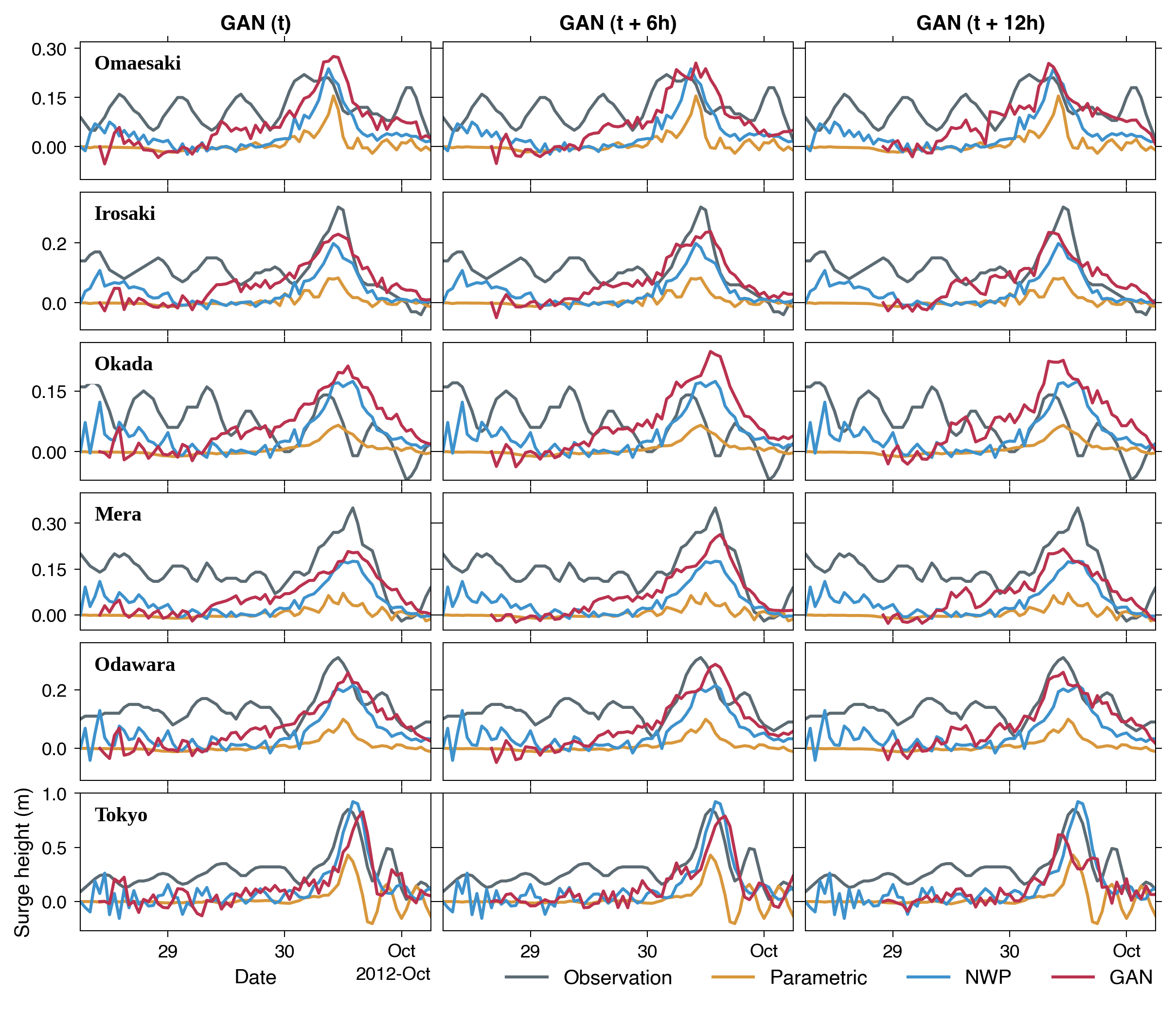
**Supplementary Fig. 18.** Same with Supplementary Fig. 16 for the 2012 Typhoon Jelawat.
