# Supplementary figures and images for: A novel deep learning approach for typhoon-induced storm surge modeling through efficient emulation of wind and pressure fields
Source: Sci Rep. 2023 May 16;13:7918. doi: 10.1038/s41598-023-35093-9 (PMC10188603; doi:10.1038/s41598-023-35093-9)

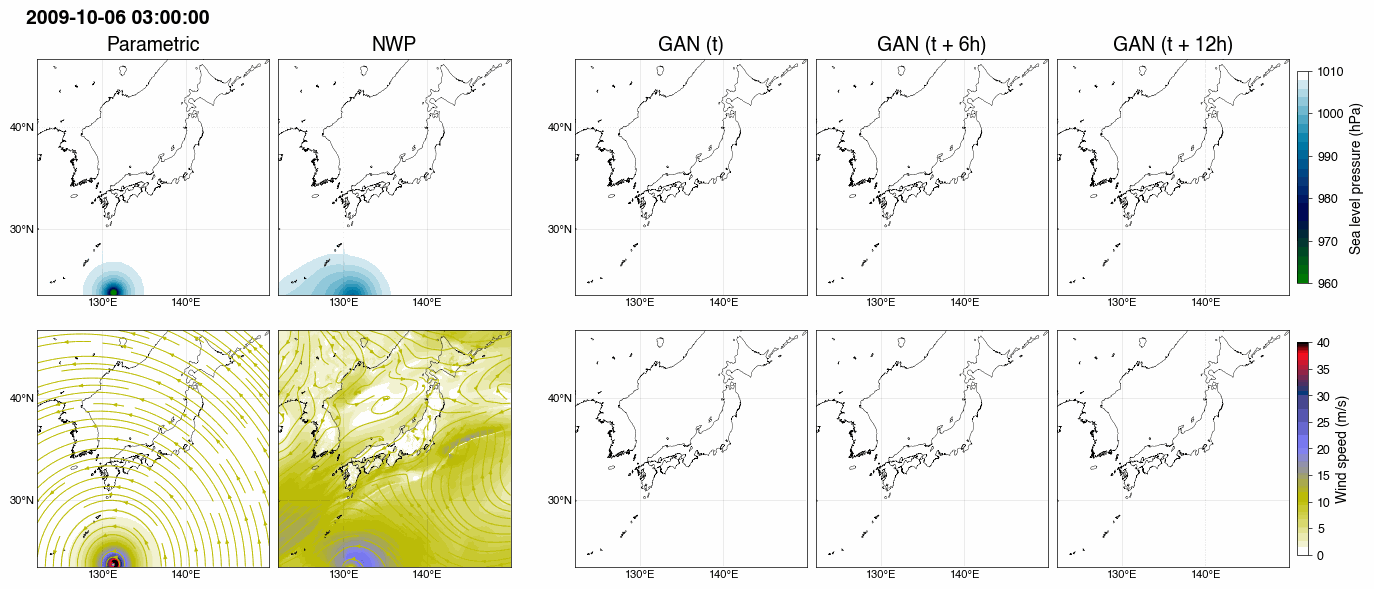

Supplement: Supplementary file 2 — Supplementary Video 1. [file 41598_2023_35093_MOESM2_ESM.gif]

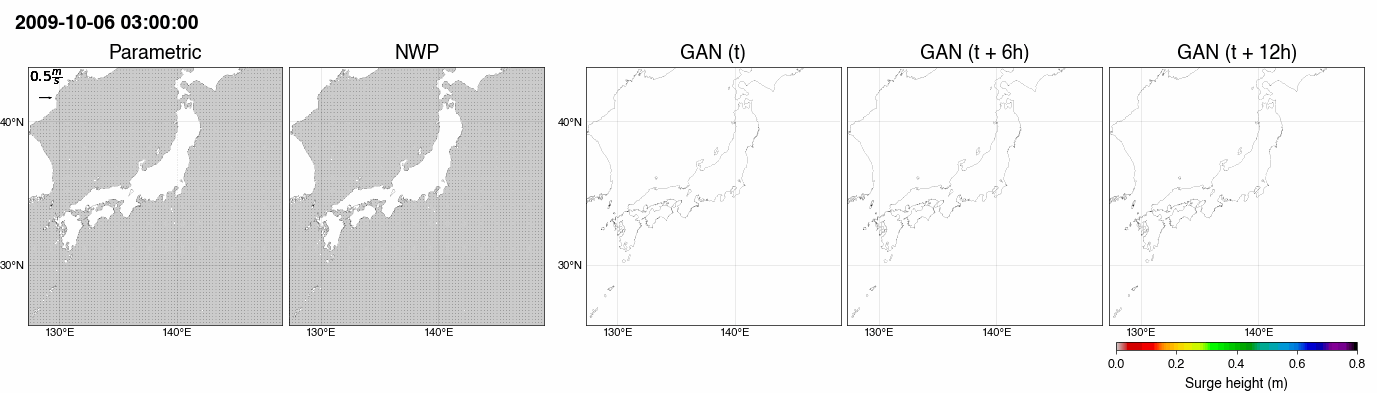

Supplement: Supplementary file 3 — Supplementary Video 2. [file 41598_2023_35093_MOESM3_ESM.gif]
